# Supplementary figures and images for: Distinguishing classes of neuroactive drugs based on computational physicochemical properties and experimental phenotypic profiling in planarians
Source: PLoS One. 2025 Jan 30;20(1):e0315394. doi: 10.1371/journal.pone.0315394 (PMC11781733; doi:10.1371/journal.pone.0315394)

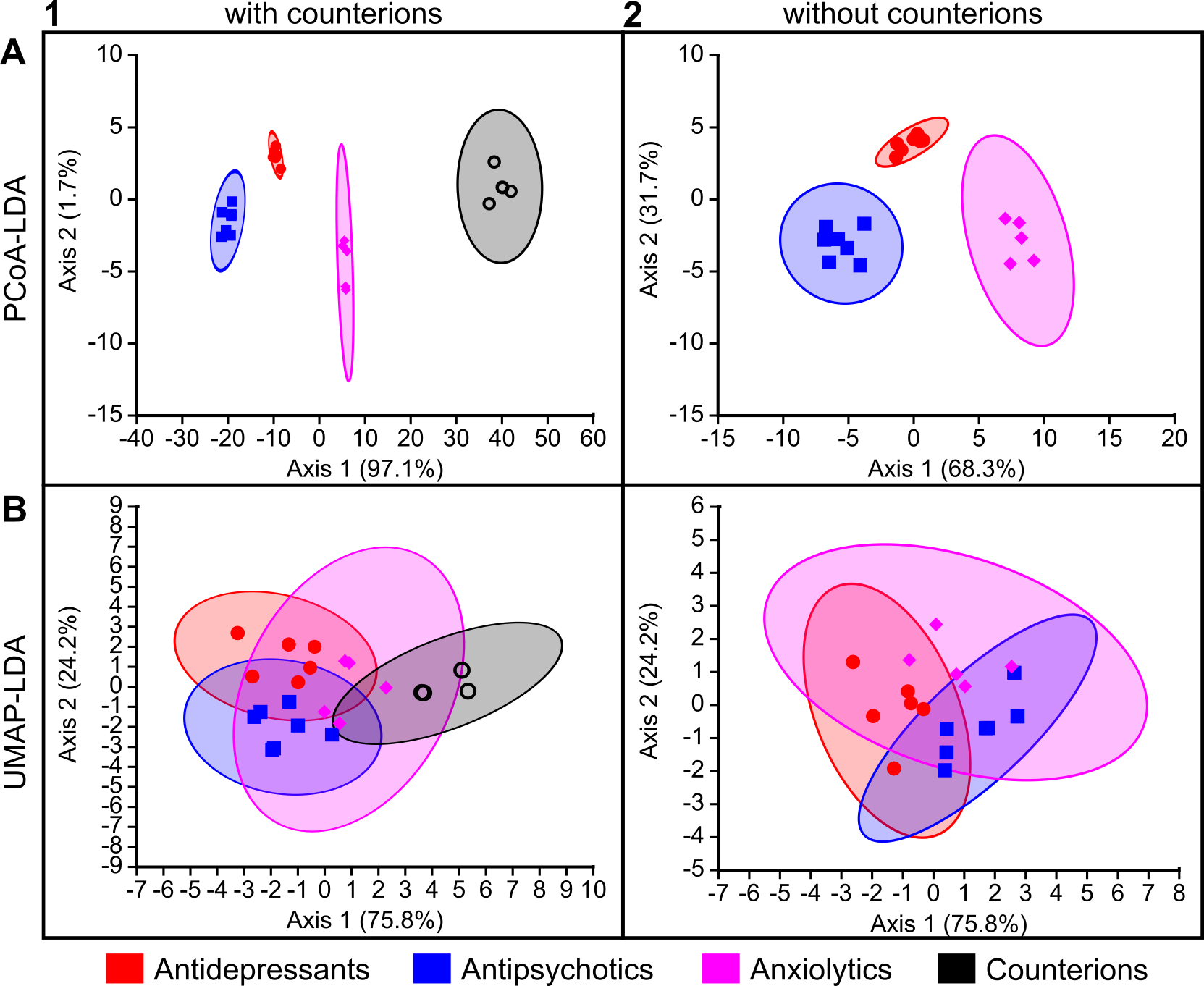

Supplement: S1 Fig — Computational methods are arranged by rows: (A) PCoA-LDA; (B) UMAP-LDA. Counterion inclusion/exclusion is arranged by columns: (1) with counterions; (2) without counterions. Misclassifications after jackknifing: (A) none; (B) PRO, BUS; (C) ARI, BUS, TRA; (D) ARI, BUS, TRA. Ellipses refer to 95% confidence intervals. The axes show the percentage of the total eigenvalues; this does not sum to 100% in (A1) because there was a third axis that accounted for the remaining 0.60%. (TIF) [file pone.0315394.s001.tif]

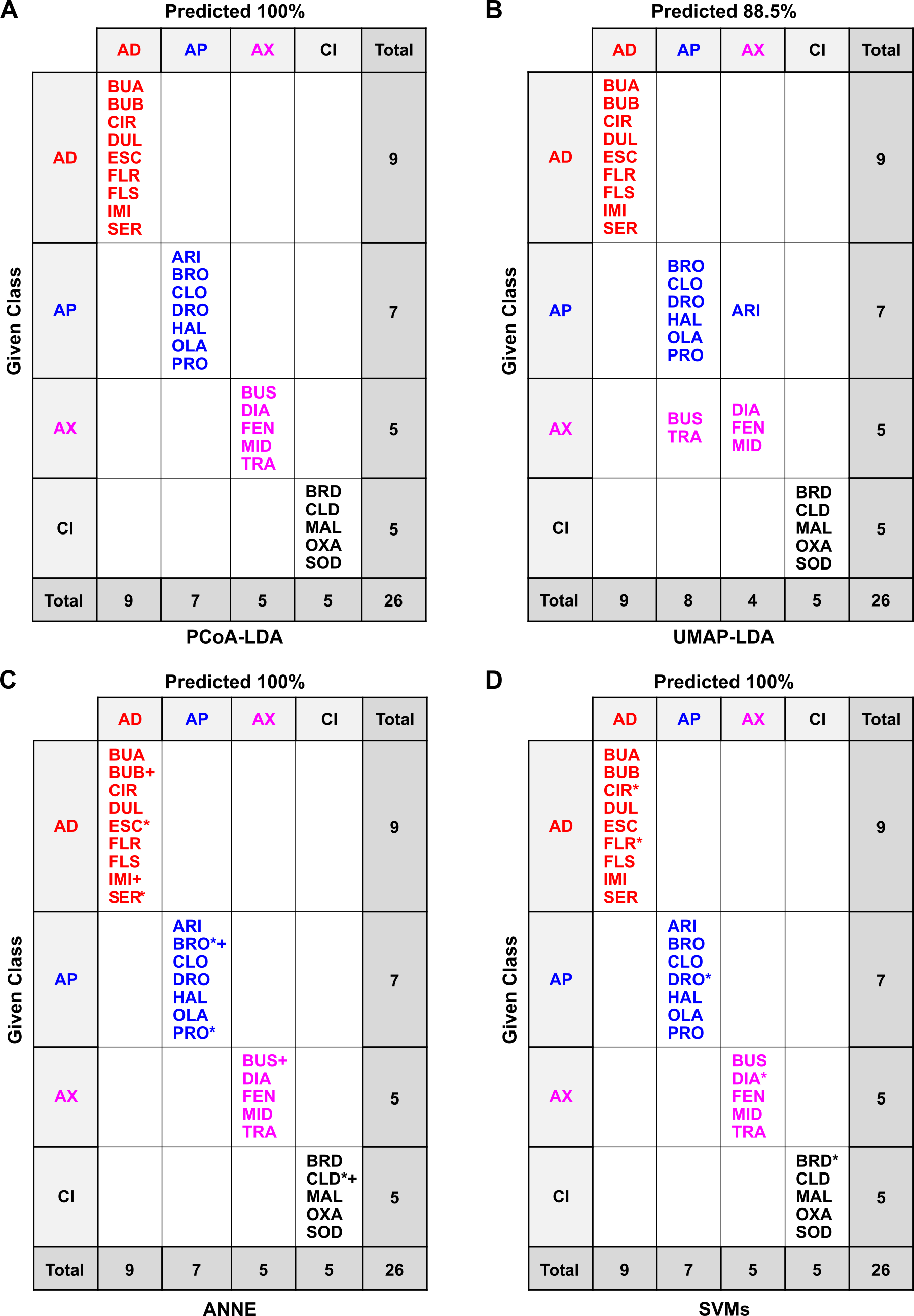

Supplement: S2 Fig — Confusion matrices for the different classification methods: (A) PCoA-LDA; (B) UMAP-LDA; (C) ANNE; (D) SVM; AD: antidepressant, AP: antipsychotic, AX: anxiolytic; CI: counterion. In A and B, predicted accuracy was calculated following an exhaustive jackknifing. In C and D, predicated accuracy refers to the overall accuracy. *indicates randomly chosen members of the test set. In (C), model 05_1n7, marked with *, was ranked first but did not include a member of the anxiolytic class in the test set. Thus, we also included the second-place model (model 06_1n8), marked with +, which included at least one member from each of the 4 classes. In (D), four models were tied for the best model but for clarity only the first of the 4 tied models is indicated because there were no misclassifications of any test set members. The members of the 4 test sets for the 4 tied first-place models were as follows: 01_2i: CIR, FLR, DRO, DIA, BRD; 05_21: ESC, FLS, BRO, MID, MAL; 06_2i: BUA, IMI, DRO, PRO, SOD; 08_21: BUA, FLS, BRO, FEN, MAL. Test set accuracies were 100% for ANNE (S6 Table) and 100% for SVMs (S8 Table). (TIF) [file pone.0315394.s002.tif]

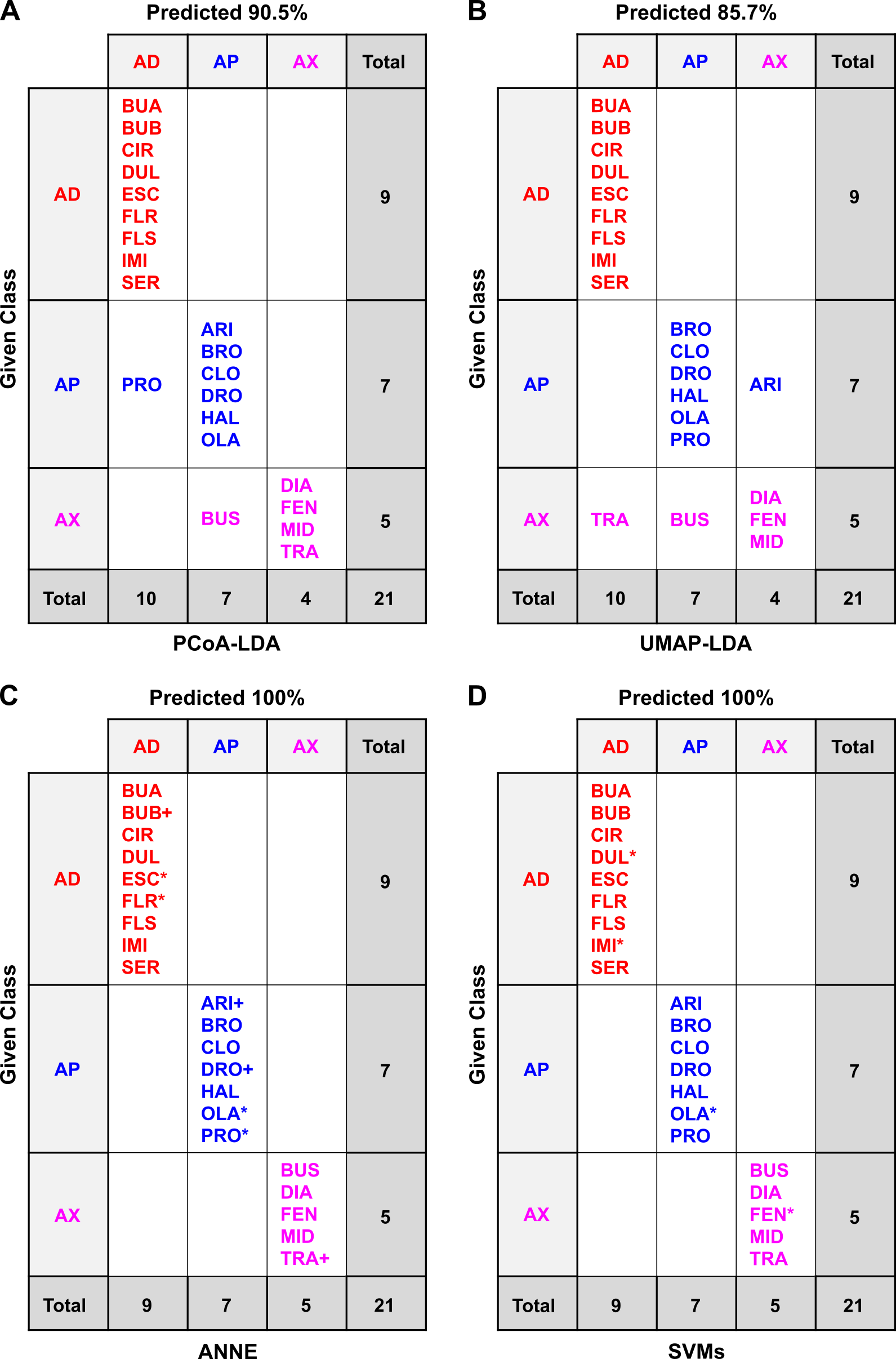

Supplement: S3 Fig — Confusion matrices for the different classification methods: (A) PCoA-LDA; (B) UMAP-LDA; (C) ANNE; (D) SVM; AD: antidepressant, AP: antipsychotic, AX: anxiolytic. In A and B, predicted accuracy was calculated following an exhaustive jackknifing. In C and D, predicted accuracy refers to the overall accuracy. *indicates randomly chosen members of the test set. In (C), model 03_1n7, marked with *, was ranked first but did not include a member of the anxiolytic class in the test set. Thus, we also included one of the second-place models (model04_2n2), marked with +, which included at least one member from each of the 4 classes. 04_2n2 was tied for second place with 09_2n2, which had the following test set members: BUA, FLS, OLA, MID. For clarity, we only marked model 04_2n2 in (C). In (D), three models were tied for the best model but for clarity only the first of the 3 tied models is indicated because there were no misclassifications of any test set members. Test set accuracies were 100% for ANNE (S7 Table) and 100% for SVMs (S9 Table). (TIF) [file pone.0315394.s003.tif]

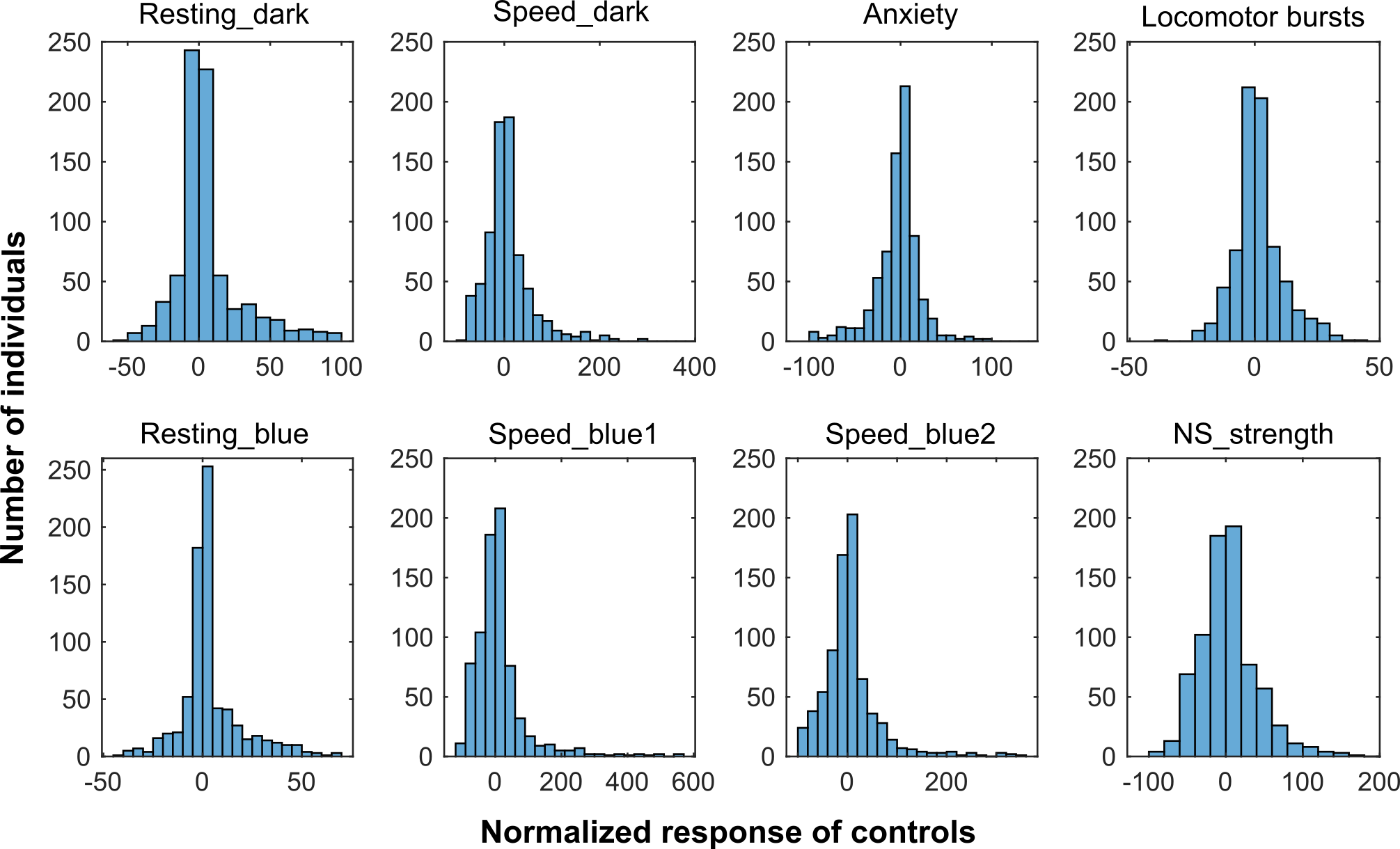

Supplement: S4 Fig — Plots show the distribution of normalized responses for each individual vehicle control (n = 768) when normalized by the median of the control population of the respective plate. (TIF) [file pone.0315394.s004.tif]

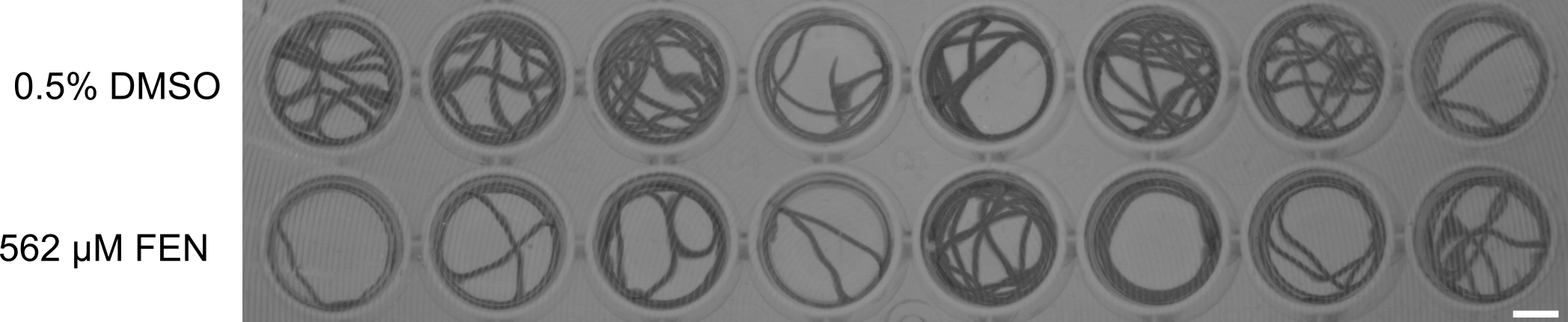

Supplement: S5 Fig — Minimum intensity projections showing the behavior of planarians exposed to either 0.5% DMSO (solvent control) or 562 μM FEN for 12 days over the course of the 5 min phototaxis assay. Images show representative worms out of a total of n = 32 per condition. Scale bar: 4 mm. (TIF) [file pone.0315394.s005.tif]

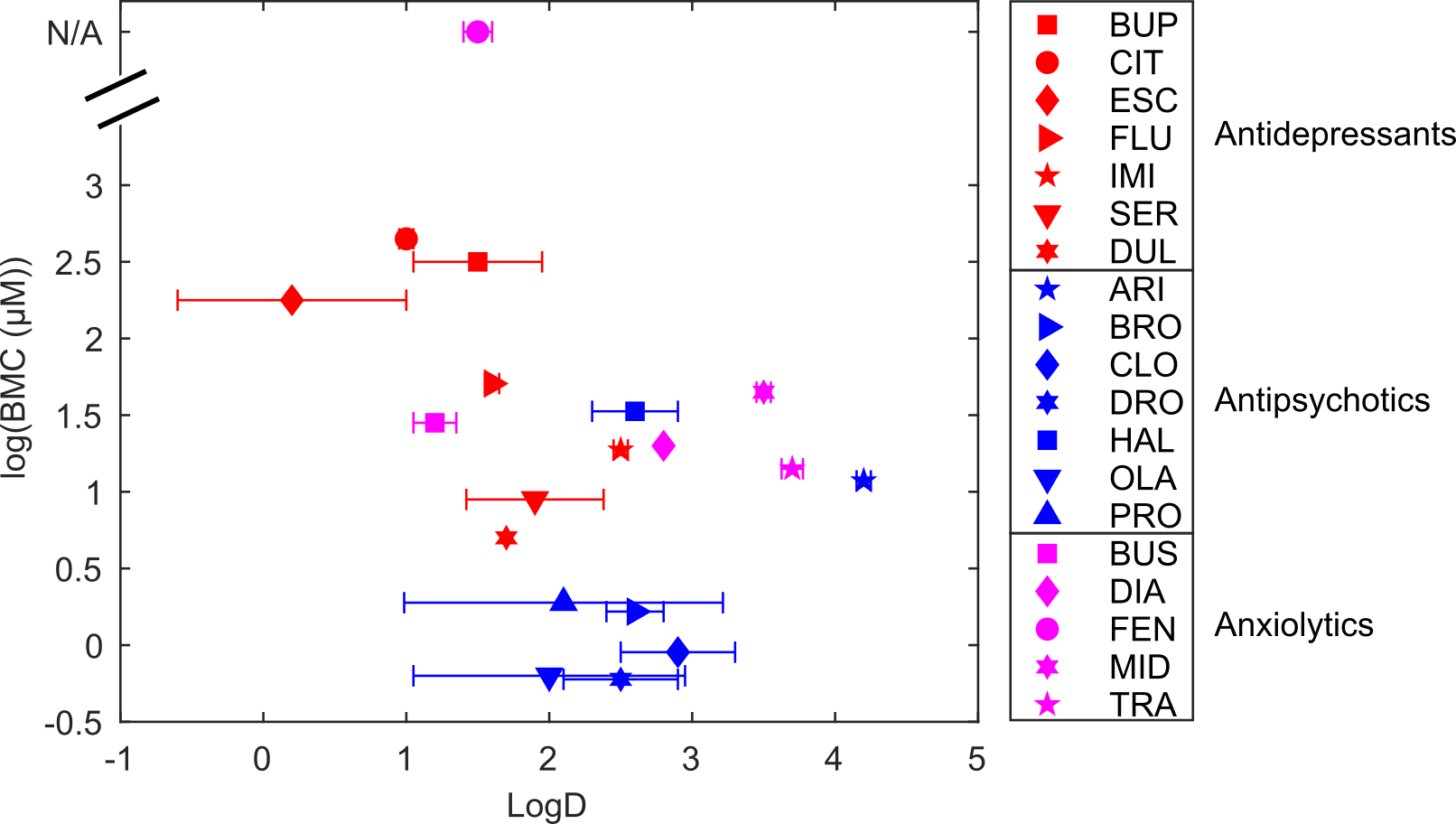

Supplement: S6 Fig — The range of logD values (error bars) for a given chemical were calculated based on the logD at the pH of the solvent and at the measured pH of the highest tested concentration (see S12 Table) to cover the suspected pH for all test concentrations and plotted against the log10 of the most sensitive benchmark concentration (BMC) in μM for that chemical. Markers were added at the median of the logD range to allow for labeling by chemical. N/A indicates that fenobam (FEN) was inactive as this did not have a calculated BMC. (TIF) [file pone.0315394.s006.tif]

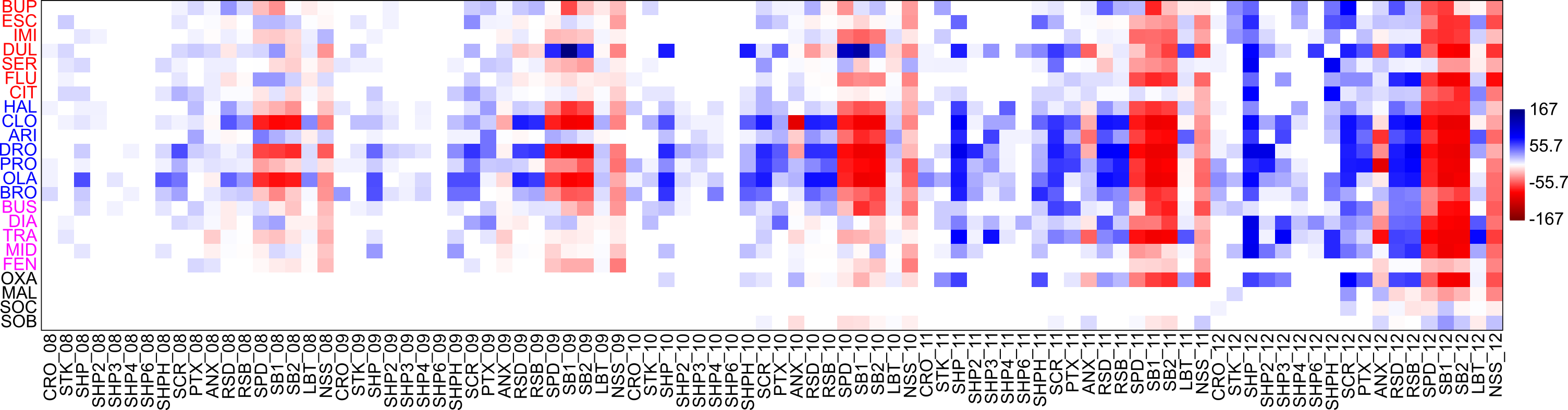

Supplement: S7 Fig — Each chemical was assigned a master barcode by concatenating the barcodes of each concentration. Only the 5 highest concentrations (relative concentrations 8–12) were used for the classification methods so that the inactive/missing data would not dominate the phenotypes. Columns are labeled as “endpoint_relative concentration”. CRO: crawl-out, STK: stickiness, SHP: body shape, SCR: scrunching, PTX: phototaxis, ANX: anxiety, RSD: resting_dark, RSB: resting_blue, SPD: speed_dark, SB1: speed_blue1, SB2: speed_blue2, LBT: locomotor bursts_total; NSS: noxious stimuli_strength. Chemicals are colored by class: antidepressants (red), antipsychotics (blue), anxiolytics (magenta), and counterions (black). (TIF) [file pone.0315394.s007.tif]

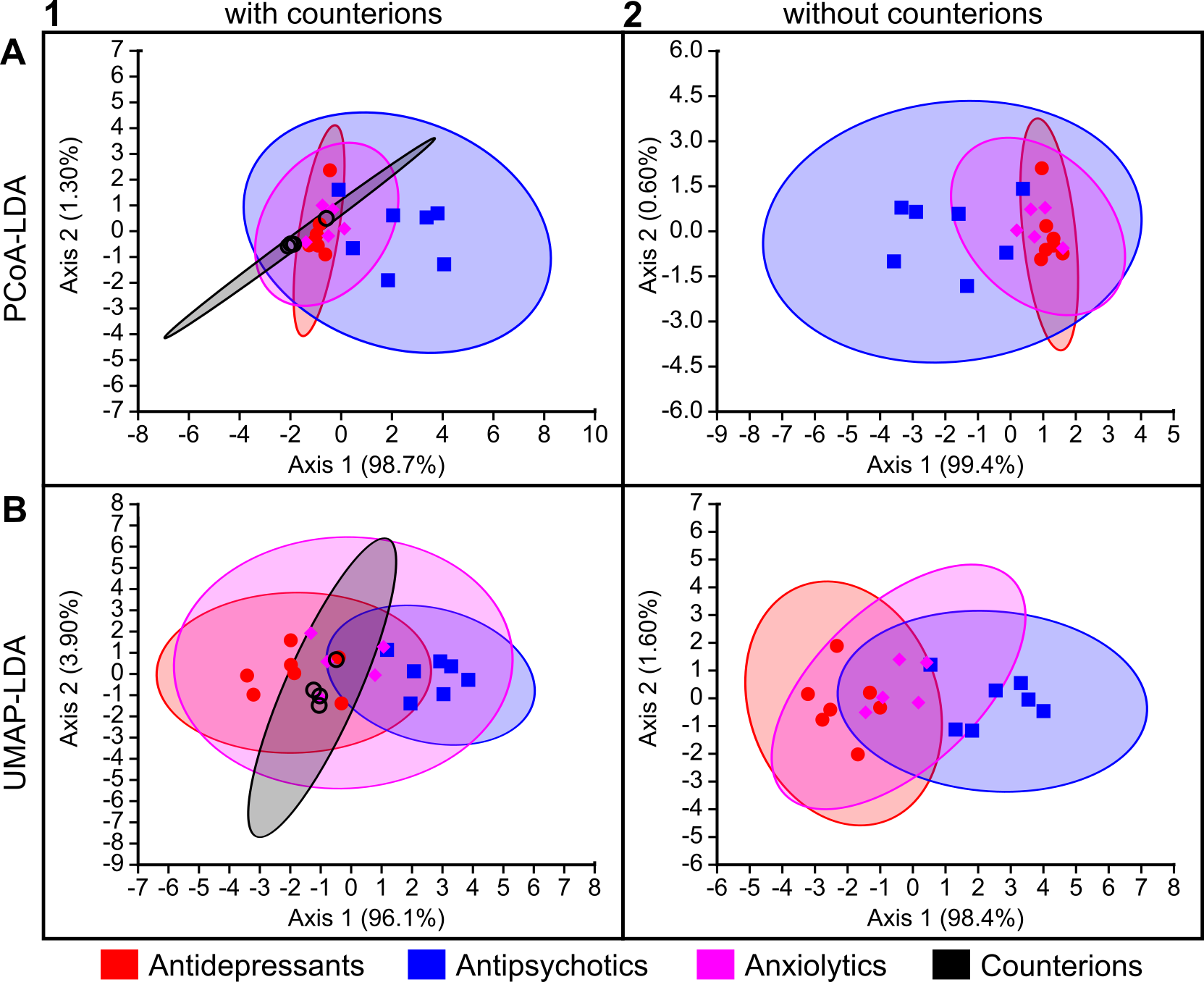

Supplement: S8 Fig — Computational methods are arranged by rows: (A) PCoA-LDA; (B) UMAP-LDA. Counterion inclusion/exclusion is arranged by columns: (1) with counterions; (2) without counterions. Ellipses show 95% confidence intervals. The axes show the percentage of the total eigenvalues. (TIF) [file pone.0315394.s008.tif]

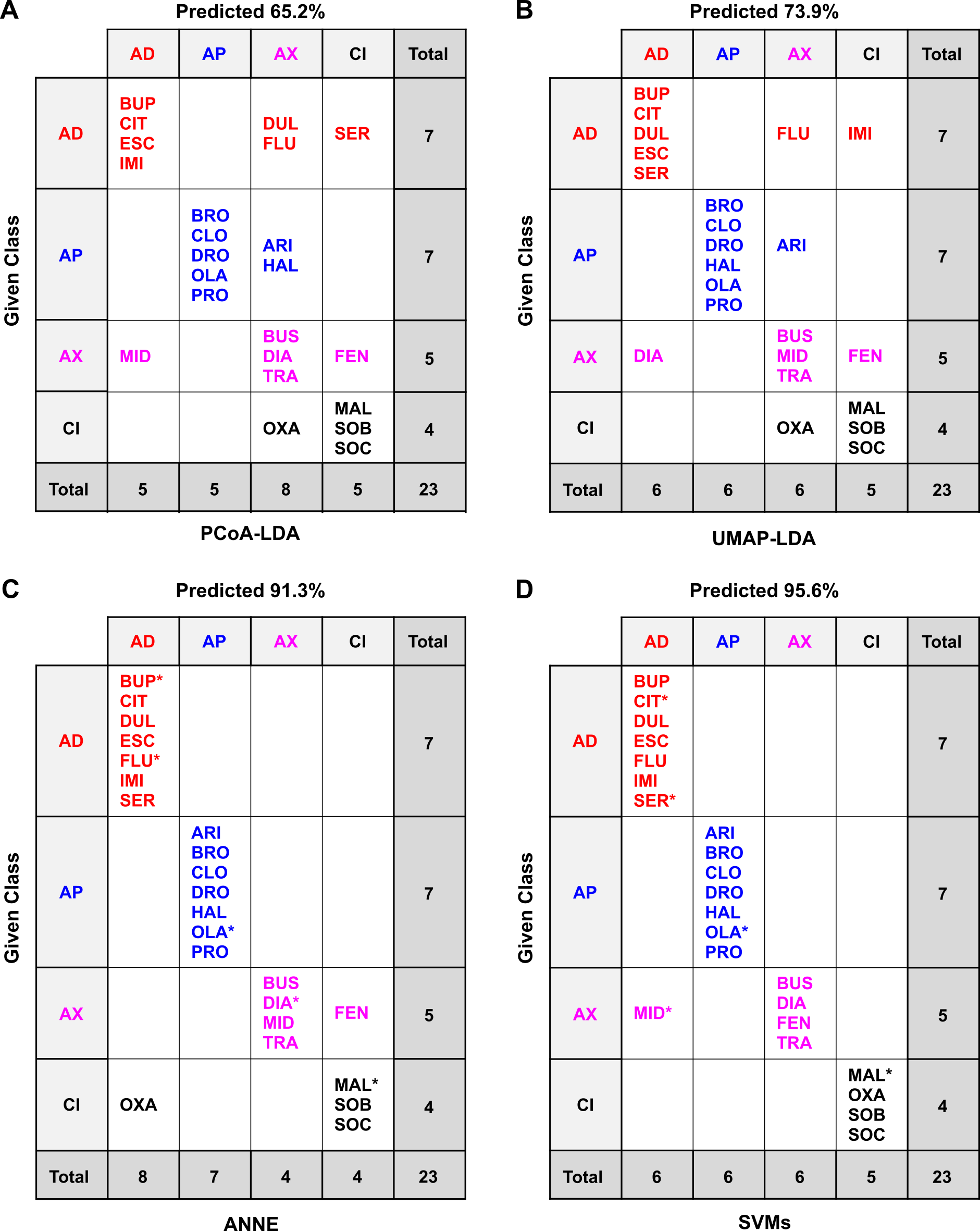

Supplement: S9 Fig — Confusion matrices for the different classification methods: (A) PCoA-LDA; (B) UMAP-LDA; (C) ANNE; (D) SVMs. AD: antidepressant, AP: antipsychotic, AX: anxiolytic; CI: counterion. In A and B, predicted accuracy was calculated following an exhaustive jackknifing. In C and D, predicated accuracy refers to the overall accuracy. *indicates randomly chosen members of the test set. Test set accuracies were 100% for ANNE (S19 Table) and 80% for SVMs (S21 Table). (TIF) [file pone.0315394.s009.tif]

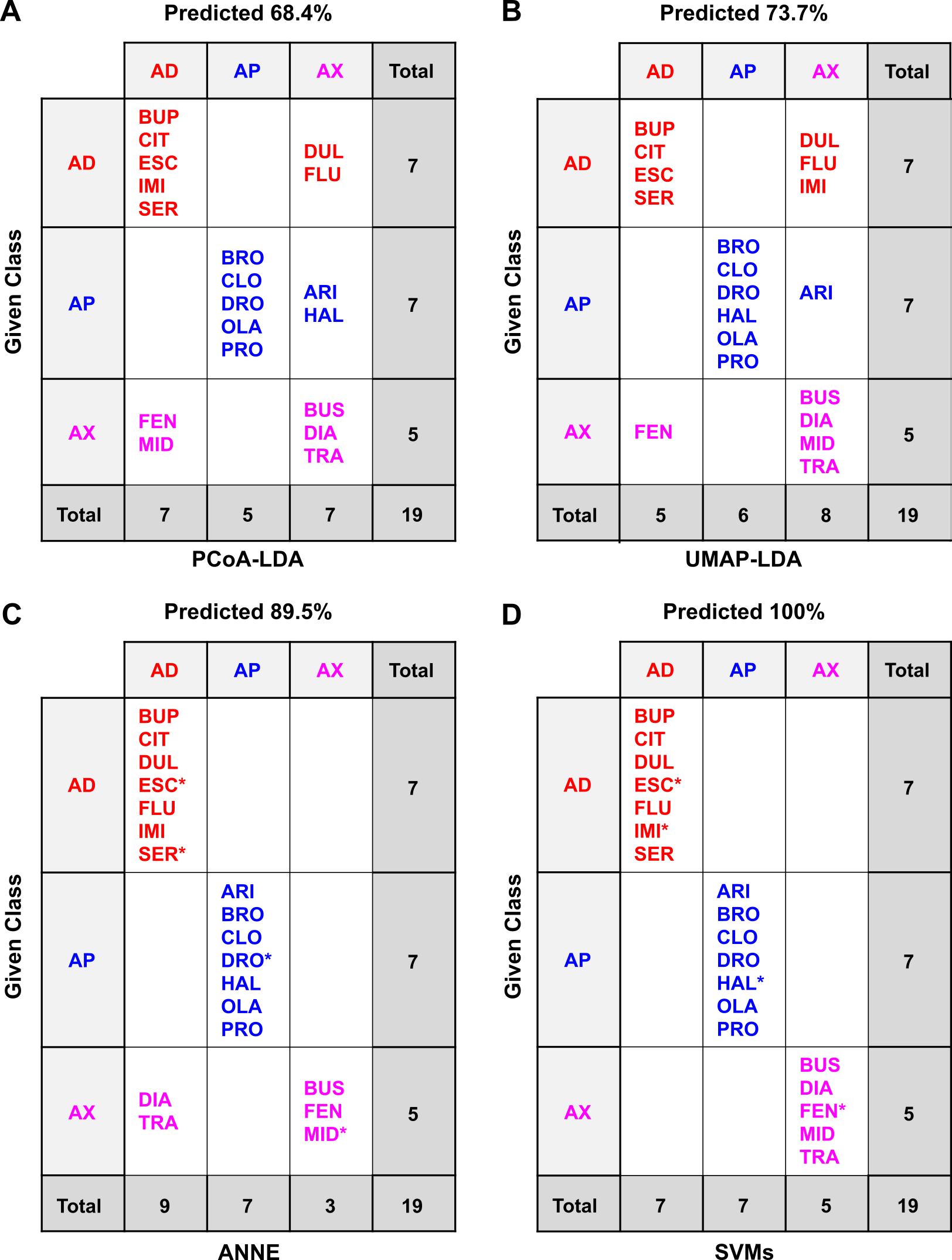

Supplement: S10 Fig — Confusion matrices for the different classification methods: (A) PCoA-LDA; (B) UMAP-LDA; (C) ANNE; (D) SVMs. AD: antidepressant, AP: antipsychotic, AX: anxiolytic. In A and B, predicted accuracy was calculated following an exhaustive jackknifing. In C and D, predicated accuracy refers to the overall accuracy. *indicates randomly chosen members of the test set. Test set accuracies were 100% for ANNE (S20 Table) and 100% for SVMs (S22 Table). (TIF) [file pone.0315394.s010.tif]
