# Supplementary material for: Distinguishing classes of neuroactive drugs based on computational physicochemical properties and experimental phenotypic profiling in planarians
Source: PLoS One. 2025 Jan 30;20(1):e0315394. doi: 10.1371/journal.pone.0315394 (PMC11781733; doi:10.1371/journal.pone.0315394)
Supplement: S1 Table — (PDF) [file pone.0315394.s011.pdf]

S1 Table. Chemical descriptors used in the machine learning models (ANNE and SVMs).

Descriptor	Definition (ADMET_Predictor_11_Manual)	Frequency
ABSQon	Sum of absolute values of partial atomic charges on O and N atoms	1
Blbn_J	Balaban distance connectivity index J of the H-suppressed molecular graph	2
EEM_Afon	Sum of absolute values of sigma Fukui indices on N and O	1
EEM_F2	Second component of the autocorrelation vector of sigma Fukui indices	1
EEM_F6	Sixth component of the autocorrelation vector of sigma Fukui indices	1
F_AFRBWF	Average value of the freely rotatable bond weight factor	4
FCation	Cumulative contribution of purely cationic species to fractioniIonized at pH 7.4	2
FUnion	Cumulative contribution of all species with zero formal charge to fraction ionized at pH 7.4	2
HBAch	Sum of estimated natural population analysis (NPA) partial atomic charges on H-bond acceptors	3
HBDnch	Sum of estimated NPA partial atomic charges on N-based H-bond donor-H	1
Key_018	[C;D2]1[C;D2][N;D3]([C;D2][C;D2]N1)[c;D3](:[c;D2]:c):[c;D3](:c)Cl	1
Key_019	C[C;D2][N;D3]([C;D2]C)[c;D3](:c):c	1
Key_028	C[N;D3](C)C	1
Key_104	c:[c;D3](:c)[F;D1]	1
Key_118	C[C;D2][C;D2][N;D2][C;D1]	1
Key_222	c1:[c;D2]:[c;D2]:[c;D2]:[c;D3](:[c;D3]:1Cl)[Cl;D1]	1
M_NO	Total number of N and O atoms	2
M_POL	Number of aromatic polar substituents excluding Ar-CX2- and Ar-CX=C<, X = C or H	5
M_PRX	Proximity effect of N and O	2
N_AliphR	Number of aliphatic rings	1
N_Pisyms	Number of distinct pi-systems, excluding lone pairs	1
NPA_AQon	Sum of absolute values of estimated NPA partial atomic charges on O and N atoms	1
NPA_Q2	Second component of the autocorrelation vector of estimated NPA partial atomic charges	1
PEoEDIIa3D	Proximity effects of electron donors of type II including atoms with hydrogens	1
Pi_ABSQ	Sum of absolute values of Hückel pi atomic charges	1
Pi_AQn	Sum of absolute values of Hückel pi atomic charges on N atoms	1
Pi_FMi5	Fifth component of the autocorrelation vector of pi Fukui_1_ indices	1
Pi_FPl1	First component of the autocorrelation vector of pi Fukui(+) indices	1
Pi_FPl5	Fifth component of the autocorrelation vector of pi Fukui (+) indices	1
SecAmine_>NH	Number of primary and aliphatic N secondary amines	1
SHaaCH	Atom-type H E-state index for aromatic (aCHa) groups	1
SHCH_321	Atom-type H E-state index for -CH3, -CH2-, and >CH- groups	1
SHssNH	Atom type hydrogen E-state for -NH- groups	1
SsCH3	Atom-type E-state index for -CH3 groups	2
T_HydroR	Topological equivalent of HydroR_3D	1
TerAmine_>N-	Number of tertiary amine groups	1
Wiener	Classical Wiener index of the H-suppressed molecular graph	1
