# Supplementary material for: Distinguishing classes of neuroactive drugs based on computational physicochemical properties and experimental phenotypic profiling in planarians
Source: PLoS One. 2025 Jan 30;20(1):e0315394. doi: 10.1371/journal.pone.0315394 (PMC11781733; doi:10.1371/journal.pone.0315394)
Supplement: S2 Table — (PDF) [file pone.0315394.s012.pdf]

S2 Table. ANNE classification models using 2D molecular descriptors of 18 drugs and 5 counterions.

rank	model	you all	mcc all	acc all	you tra	mcc tra	acc tra	you tes	mcc tes	acc tes	mis	obs	pred
3	01_1n7	93.9	94.4	95.7	100	100	100	66.7	75.6	80.0	SOD	3	0
8	02_1n2	69.0	72.8	78.3	75.8	78.5	83.3	50.0	56.7	60.0	CIT<br>BUS<br>FEN<br>IMI<br>TRA	0<br>2<br>2<br>0<br>2	1<br>1<br>0<br>1<br>1
**1**	**03_1n6**	**93.9**	**94.4**	**95.6**	**100**	**100**	**100**	**77.8**	**77.8**	**80.0**	**IMI**	**0**	**1**
10	04_1n8	81.7	83.0	87.0	91.7	92.8	94.4	50.0	50.0	60.0	TRA<br>BUP<br>MID	2<br>0<br>2	1<br>2<br>1
7	05_1n5	82.5	82.7	87.0	100	100	100	16.7	17.7	40.0	BUP<br>PRO<br>DIA	0<br>1<br>2	3<br>0<br>1
5	06_1n5	88.1	88.7	91.3	100	100	100	50.0	53.0	60.0	BUP<br>TRA	0<br>2	1<br>3
9	07_2n1	69.0	74.1	78.3	68.3	73.4	77.8	66.7	75.6	80.0	BUS<br>DIA<br>FEN	2<br>2<br>2	0<br>1<br>0
4	08_1n3	81.7	83.0	87.0	84.2	85.2	88.9	72.2	76.6	80.0	BUP<br>MID<br>DIA	0<br>2<br>2	2<br>1<br>1
2	09_1n6	93.7	94.4	95.7	100	100	100	72.2	76.6	80.0	BUS	2	1
6	10_1n5	70.1	71.1	78.3	69.4	71.2	77.8	77.8	77.8	80.0	IMI<br>HAL<br>DIA<br>FEN<br>BRO	0<br>1<br>2<br>2<br>1	1<br>0<br>1<br>1<br>0
Mean ± SEM (*n* = 10)		82.4 ± 3.22	83.9 ± 2.87	87.4 ± 2.29	88.9 ± 4.24	90.1 ± 3.79	92.2 ± 3.01	60.1 ± 5.96	63.7 ± 6.22	70.0 ± 4.47	NA	NA	NA

ANNE, artificial neural network ensemble; model (e.g., 1n7, 1 neuron and 7 descriptors); you, Youden index; mcc, Matthews correlation coefficient; acc, accuracy; all, combined score for training and test sets; tra. training set, tes, test set; mis, misclassified drug or counterion; obs, observed class; pred, predicted class; classes: 0, antidepressant (red); 1, antipsychotic (blue); 2, anxiolytic (magenta); 3, counterion (gray). NA, not applicable. Statistical scores are expressed as percentages and defined in the Methods. Each model was started with a different random seed number and a training:test ratio of 18:5 compounds. Test set partition: stratified by CLASS using random selection. The three-letter code names for the drugs are given in Table 1. The top-ranked model (shown in bold) used the following descriptors and relative sensitivities: N_Pisyms (1.000), NPA_Q2 (0.999), SecAmine_>NH (0.763), Pi_AFPIl (0.726), M_PRX (0.722), and M_POL (0.698); random seed = 72201. Chemical descriptor definitions are listed in S1 Table. The rank for each model was determined by applying the RANK.AVG function in Microsoft Excel 365 to SUM(training metrics + test metrics + $(100\times N_{min}/N)$ + $(100\times D_{min})$ $/D)$), where N_{min} = minimum number of neurons, N = number of neurons, D_{min} = minimum number of descriptors, and D = number of descriptors.
