# Supplementary material for: Distinguishing classes of neuroactive drugs based on computational physicochemical properties and experimental phenotypic profiling in planarians
Source: PLoS One. 2025 Jan 30;20(1):e0315394. doi: 10.1371/journal.pone.0315394 (PMC11781733; doi:10.1371/journal.pone.0315394)
Supplement: S3 Table — (PDF) [file pone.0315394.s013.pdf]

S3 Table. ANNE classification models using 2D molecular descriptors of 18 drugs.

rank	model	you all	mcc all	acc all	you tra	mcc tra	acc tra	you tes	mcc tes	acc tes	mis	obs	pred
**1**	**01_1n4**	**100**	**100**	**100**	**100**	**100**	**100**	**100**	**100**	**100**	**NA**	**NA**	**NA**
9	02_1n6	84.6	84.6	88.9	100	100	100	30.0	38.7	50.0	CIT<br>DRO	0<br>1	2<br>2
3	03_1n4	92.1	92.1	94.4	100	100	100	70.0	70.0	75.0	DUL	0	2
4	04_1n4	92.1	92.1	94.4	100	100	100	50.0	64.5	75.0	OLA	1	0
6	05_1n1	83.2	83.2	88.9	100	100	100	20.0	20.0	50.0	BUP<br>OLA	0<br>1	1<br>0
5	06_1n4	84.6	84.6	89.9	89.2	89.9	92.9	70.0	70.0	75.0	PRO<br>DRO	1<br>1	0<br>2
8	07_1n3	82.7	83.5	89.9	100	100	100	30.0	30.0	50.0	DUL<br>BUS	0<br>2	2<br>1
7	08_1n2	83.2	83.2	89.9	87.5	89.6	92.9	50.0	64.5	25.0	BUP<br>OLA	0<br>1	1<br>0
10	09_1n3	76.2	76.2	83.3	90.0	90.0	92.9	20.0	22.4	50.0	DRO<br>CIT<br>ARI	1<br>0<br>1	2<br>1<br>2
2	10_1n2	90.7	92.0	94.4	100	100	100	60.0	67.1	75.0	MID	2	1
Mean ± SEM (n = 10)		86.9 ± 2.13	87.2 ± 2.15	91.4 ± 1.44	96.7 ± 1.71	97.0 ± 1.55	97.9 ± 1.08	50.0 ± 8.16	54.7 ± 8.15	62.5 ± 6.72	NA	NA	NA

ANNE, artificial neural network ensemble; model (e.g., 1n4, 1 neuron and 4 descriptors); you, Youden index; mcc, Matthews correlation coefficient; acc, accuracy; all, combined score for training and test sets; tra. training set, tes, test set; mis, misclassified drug; obs, observed class; pred, predicted class; classes: 0, antidepressant (red); 1, antipsychotic (blue); 2, anxiolytic (magenta); NA, not applicable. Statistical scores are expressed as percentages and defined in the Methods. Each model was started with a different random seed number and a training:test ratio of 14:4 compounds. Test set partition: stratified by CLASS using random selection. The three-letter code names for the drugs are given in Table 1. The top-ranked model (shown in bold) used the following descriptors and relative sensitivities: Key_18 (1.000), M_PRX (0.998), HBDnch (0.995), and Key_19 (0.984); random seed = 11755. Chemical descriptor definitions are listed in S1 Table. The rank for each model was determined by applying the RANK.AVG function in Microsoft Excel 365 to SUM(training metrics + test metrics + $(100\times N_{min}/N)$ + $(100\times D_{min})$ $/D)$), where N_{min} = minimum number of neurons, N = number of neurons, D_{min} = minimum number of descriptors, and D = number of descriptors.
