# Supplementary material for: Distinguishing classes of neuroactive drugs based on computational physicochemical properties and experimental phenotypic profiling in planarians
Source: PLoS One. 2025 Jan 30;20(1):e0315394. doi: 10.1371/journal.pone.0315394 (PMC11781733; doi:10.1371/journal.pone.0315394)
Supplement: S4 Table — (PDF) [file pone.0315394.s014.pdf]

S4 Table. SVMs classification models using 2D molecular descriptors of 18 drugs and 5 counterions.

rank	model	you all	mcc all	acc all	you tra	mcc tra	acc tra	you tes	mcc tes	acc tes	mis	obs	pred
3.5	01_4i	100	100	100	100	100	100	100	100	100	NA	NA	NA
10	02_10i	87.8	89.2	91.3	100	100	100	55.6	63.0	60.0	CIT<br>IMI	0<br>0	1<br>1
6.5	03_2i	93.9	94.4	95.7	100	100	100	66.7	75.6	80.0	SOD	3	0
8	04_10i	100	100	100	100	100	100	100	100	100	NA	NA	NA
5	05_6i	100	100	100	100	100	100	100	100	100	NA	NA	NA
9	06_12i	100	100	100	100	100	100	100	100	100	NA	NA	NA
**1.5**	**07_2i**	**100**	**100**	**100**	**100**	**100**	**100**	**100**	**100**	**100**	**NA**	**NA**	**NA**
6.5	08_2i	93.9	94.4	95.7	100	100	100	66.7	75.6	80.0	BUS	2	0
**1.5**	**09_2i**	**100**	**100**	**100**	**100**	**100**	**100**	**100**	**100**	**100**	**NA**	**NA**	**NA**
3.5	10_4i	100	100	100	100	100	100	100	100	100	NA	NA	NA
Mean ± SEM (*n* = 10)		97.6 ± 1.35	97.8 ± 1.21	98.3 ± 0.96	100 ± 0.00	100 ± 0.00	100 ± 0.00	88.9 ± 5.73	91.4 ± 4.50	92.0 ± 4.42	NA	NA	NA

SVMs, support vector machines; model (e.g., 4i, 4descriptors); you, Youden index; mcc, Matthews correlation coefficient; acc, accuracy; all, combined score for training and test sets; tra. training set, tes, test set; mis, misclassified drug or counterion; obs, observed class; pred, predicted class; classes: 0, antidepressant (red); 1, antipsychotic (blue); 2, anxiolytic (magenta); 3, counterion (gray). NA, not applicable. Statistical scores are expressed as percentages and defined in the Methods. Each model was started with a different random seed number and a training:test ratio of 18:5 compounds. Test set partition: stratified by CLASS using random selection. The three-letter code names for the drugs are given in Table 1. The two top-ranked models (shown in bold) used the following descriptors and relative sensitivities: 07_2i, M_POL (1.000), M_NO (0.995), random seed = 99041; 09_2i, F_AFRBWF (1.000), HBAch (0.948), random seed = 96552. Chemical descriptor definitions are listed in S1 Table.
