# Supplementary material for: Distinguishing classes of neuroactive drugs based on computational physicochemical properties and experimental phenotypic profiling in planarians
Source: PLoS One. 2025 Jan 30;20(1):e0315394. doi: 10.1371/journal.pone.0315394 (PMC11781733; doi:10.1371/journal.pone.0315394)
Supplement: S5 Table — (PDF) [file pone.0315394.s015.pdf]

S5 Table. SVMs classification models using 2D molecular descriptors of 18 drugs.

rank	model	you all	mcc all	acc all	you tra	mcc tra	acc tra	you tes	mcc tes	acc tes	mis	obs	pred
5.5	01_6i	100	100	100	100	100	100	100	100	100	NA	NA	NA
5.5	02_6i	100	100	100	100	100	100	100	100	100	NA	NA	NA
4	03_5i	100	100	100	100	100	100	100	100	100	NA	NA	NA
7	04_2i	91.1	92.0	94.4	100	100	100	50.0	64.5	75.0	TRA	2	0
9	05_4i	90.7	92.0	94.4	100	100	100	60.0	67.1	75.0	BUS	2	1
**1**	**06_2i**	**100**	**100**	**100**	**100**	**100**	**100**	**100**	**100**	**100**	**NA**	**NA**	**NA**
2	07_3i	100	100	100	100	100	100	100	100	100	NA	NA	NA
8	08_3i	92.1	92.1	94.4	100	100	100	70.0	70.0	75.0	OLA	1	0
3	09_4i	100	100	100	100	100	100	100	100	100	NA	NA	NA
10	10_6i	91.1	92.0	94.4	100	100	100	50.0	64.5	75.0	DUL	0	1
Mean ± SEM (*n* = 10)		96.5 ± 1.43	96.8 ± 1.30	97.8 ± 0.91	100 ± 0.00	100 ± 0.00	100 ± 0.00	83.0 ± 7.16	86.6 ± 5.49	90.0 ± 4.08	NA	NA	NA

SVMs, support vector machines; model (e.g., 6i, 6 descriptors); you, Youden index; mcc, Matthews correlation coefficient; acc, accuracy; all, combined score for training and test sets; tra. training set, tes, test set; mis, misclassified drug; obs, observed class; pred, predicted class; classes: 0, antidepressant (red); 1, antipsychotic (blue); 2, anxiolytic (magenta). NA, not applicable. Statistical scores are expressed as percentages and defined in the Methods. Each model was started with a different random seed number and a training:test ratio of 14:4 compounds. Test set partition: stratified by CLASS using random selection. The three-letter code names for the drugs are given in Table 1. The top-ranked model (shown in bold) used the following descriptors and relative sensitivities: M_NO (1.000), M_POL (0.990); random seed = 4211. Chemical descriptor definitions are listed in S1 Table. The three-letter code names for the drugs are given in Table 1. The rank for each model was determined by applying the RANK.AVG function in Microsoft Excel 365 to SUM(training metrics + test metrics + (100×$D_{min)}$ /D)), where D_{min} = minimum number of descriptors, and D = number of descriptors.
