# Supplementary material for: Distinguishing classes of neuroactive drugs based on computational physicochemical properties and experimental phenotypic profiling in planarians
Source: PLoS One. 2025 Jan 30;20(1):e0315394. doi: 10.1371/journal.pone.0315394 (PMC11781733; doi:10.1371/journal.pone.0315394)
Supplement: S6 Table — (PDF) [file pone.0315394.s016.pdf]

S6 Table. ANNE classification models using 3D molecular descriptors of 21 drugs and 5 counterions.

rank	model	you all	mcc all	acc all	you tra	mcc tra	acc tra	you tes	mcc tes	acc tes	mis	obs	pred
9	01_2n2	85.5	85.0	88.5	88.1	87.3	90.5	77.8	77.8	80.0	IMI	0	1
											CLO	1	2
											OLA	1	2
8	02_2n2	88.7	89.6	92.3	93.9	93.9	95.2	62.5	72.2	80.0	BUS	2	3
											SOD	3	0
3	03_1n10	100	100	100	100	100	100	100	100	100	NA	NA	NA
6	04_1n9	94.0	94.9	96.2	100	100	100	72.2	76.6	80.0	FEN	2	0
**1**	**05_1n7**	**100**	**100**	**100**	**100**	**100**	**100**	**100**	**100**	**100**	**NA**	**NA**	**NA**
**2**	**06_1n8**	**100**	**100**	**100**	**100**	**100**	**100**	**100**	**100**	**100**	**NA**	**NA**	**NA**
10	07_1n4	84.3	84.8	88.5	93.2	93.8	95.2	44.4	47.1	60.0	ARI	1	0
											DRO	1	3
											MAL	3	2
2	08_2n2	88.7	89.6	92.3	93.2	93.8	95.2	72.2	76.6	80.0	PRO	1	0
											OXA	3	1
4	09_1n6	88.7	90.2	92.3	87.1	88.2	90.5	100	100	100	BUS	2	1
											TRA	2	1
5	10_1n3	88.7	90.2	92.3	92.5	93.7	95.2	77.8	77.8	80.0	PRO	1	0
											OLA	1	0
Mean ± SEM (n = 10)		91.9 ± 1.95	92.4 ± 1.88	94.2 ± 1.43	94.8 ± 1.58	95.1 ± 1.53	96.2 ± 1.19	80.7 ± 6.06	82.8 ± 5.47	86.0 ± 4.27	NA	NA	NA

ANNE, artificial neural network ensemble; model (e.g., 2n2, 2 neurons and 2 descriptors); you, Youden index; mcc, Matthews correlation coefficient; acc, accuracy; all, combined score for training and test sets; tra. training set, tes, test set; mis, misclassified drug or counterion; obs, observed class; pred, predicted class; classes: 0, antidepressant (red); 1, antipsychotic (blue); 2, anxiolytic (magenta); 3, counterion (gray). NA, not applicable. Statistical scores are expressed as percentages and defined in the Methods. Each model was started with a different random seed number and a training:test ratio of 21:5 compounds. Test set partition: stratified by CLASS using random selection. The three-letter code names for the drugs are given in Table 1. The top-ranked model (shown in bold) used the following descriptors and relative sensitivities: SsCH3 (1.000), Key_10 4 (0.981), SHaaCH (0.975), Wiener (0.971), TerAmine_>N- (0.971), Pi_AQn (0.970), and N_AlipR (0.967); random seed = 59252. The ranking scores of the first- and second-ranked models, 05_1n7, and 06_1n8, respectively, were extremely close (91.07 and 90.62, respectively). Model 05_1n7 did not include a member from the Anxiolytic class in its test set, whereas model 06_1n8 did. Therefore, the results from both models were included in the confusion matrix shown in S2 Fig C. The descriptors and relative sensitivities for the 8 descriptors used in model 06_1n8 were EEM_F2 (1.000), Key_118 (0.998), PEoEDIIa3D (0.998), Pi_FMi5 (0.997), Key_222 (0.997), SHssNH (0.994), Pi_FPl5 (0.795), FCation (0.788). Chemical descriptor definitions are listed in S1 Table. The rank for each model was determined by applying the RANK.AVG function in Microsoft Excel 365 to SUM(training metrics + test metrics + $(100\times N_{min}/N)$ + $(100\times D_{min}/D)$), where N_{min} = minimum number of neurons, N = number of neurons, D_{min} = minimum number of descriptors, and D = number of descriptors.
