# Supplementary material for: Distinguishing classes of neuroactive drugs based on computational physicochemical properties and experimental phenotypic profiling in planarians
Source: PLoS One. 2025 Jan 30;20(1):e0315394. doi: 10.1371/journal.pone.0315394 (PMC11781733; doi:10.1371/journal.pone.0315394)
Supplement: S7 Table — (PDF) [file pone.0315394.s017.pdf]

**S7 Table. ANNE classification models using 3D molecular descriptors of 21 drugs.**

| rank | model | you all | mcc all | acc all | you tra | mcc tra | acc tra | you tes | mcc tes | acc tes | mis | obs | pred |
|---|---|---|---|---|---|---|---|---|---|---|---|---|---|
| 9 | 01_2n2 | 83.9 | 86.4 | 90.5 | 79.8 | 83.4 | 88.2 | 100 | 100 | 100 | DIA<br>MID | 2<br>2 | 1<br>1 |
| 7 | 02_1n2 | 91.3 | 92.9 | 95.2 | 100 | 100 | 100 | 50.0 | 64.5 | 75.0 | MID | 2 | 0 |
| **1** | **03_1n7** | **100** | **100** | **100** | **100** | **100** | **100** | **100** | **100** | **100** | **NA** | **NA** | **NA** |
| 2.5 | 04_2n2 | 100 | 100 | 100 | 100 | 100 | 100 | 100 | 100 | 100 | NA | NA | NA |
| 5.5 | 05_1n1 | 86.7 | 86.7 | 90.5 | 91.5 | 91.5 | 94.1 | 70.0 | 70.0 | 75.0 | ARI<br>PRO | 1<br>1 | 2<br>0 |
| 4 | 06_2n1 | 86.7 | 86.7 | 90.5 | 83.0 | 83.9 | 88.2 | 100 | 100 | 100 | BUA<br>BUB | 0<br>0 | 1<br>1 |
| 5.5 | 07_2n2 | 92.0 | 92.9 | 95.2 | 90.4 | 91.4 | 94.1 | 100 | 100 | 100 | OLA | 1 | 0 |
| 8 | 08_1n3 | 80.1 | 80.9 | 85.7 | 76.1 | 77.3 | 82.4 | 100 | 100 | 100 | ARI<br>CLO<br>DRO | 1<br>1<br>1 | 2<br>2<br>2 |
| 2.5 | 09_2n2 | 100 | 100 | 100 | 100 | 100 | 100 | 100 | 100 | 100 | NA | NA | NA |
| 10 | 10_2n2 | 92.0 | 92.9 | 95.2 | 100 | 100 | 100 | 50.0 | 64.5 | 75.0 | BUS | 2 | 1 |
| Mean ± SEM (*n* = 10) | | 91.3 ± 2.24 | 91.9 ± 2.12 | 94.3 ± 1.55 | 92.1 ± 2.99 | 92.8 ± 2.73 | 94.7 ± 2.05 | 87.0 ± 6.84 | 89.9 ± 5.16 | 92.5 ± 3.82 | NA | NA | NA |

ANNE, artificial neural network ensemble; model (e.g., 2n1, 2 neurons and 1 descriptor); you, Youden index; mcc, Matthews correlation coefficient; acc, accuracy; all, combined score for training and test sets; tra. training set, tes, test set; mis, misclassified drug; obs, observed class; pred, predicted class; classes: 0 (red), antidepressant; 1, antipsychotic (blue); 2, anxiolytic (magenta); NA, not applicable. Statistical scores are expressed as percentages and defined in the Methods. Each model was started with a different random seed number and a training:test ratio of 17:4 compounds. Test set partition: stratified by CLASS using random selection. The three-letter code names for the drugs are given in Table 1. The top-ranked model (shown in bold) used the following descriptors and relative sensitivities: SsCH3 (1.000), Pi_ABSQ (0.997), SHCH_321 (0.994), Key_28 (0.993), ABSQond (0.992), T_HydroR (0.992), F_AFRB (0.992); random seed = 51071. Chemical descriptor definitions are listed in S1 Table. The rank for each model was determined by applying the RANK.AVG function in Microsoft Excel 365 to SUM(training metrics + test metrics + $(100\times N_{min}/N)$ + $(100\times D_{min})$ $/D)$), where $N_{min}$ = minimum number of neurons, $N$ = number of neurons, $D_{min}$ = minimum number of descriptors, and $D$ = number of descriptors.
