# Supplementary material for: Distinguishing classes of neuroactive drugs based on computational physicochemical properties and experimental phenotypic profiling in planarians
Source: PLoS One. 2025 Jan 30;20(1):e0315394. doi: 10.1371/journal.pone.0315394 (PMC11781733; doi:10.1371/journal.pone.0315394)
Supplement: S8 Table — (PDF) [file pone.0315394.s018.pdf]

S8 Table. SVMs classification models using 3D molecular descriptors of 21 drugs and 5 counterions.

rank	model	you all	mcc all	acc all	you tra	mcc tra	acc tra	you tes	mcc tes	acc tes	mis	obs	pred
**2.5**	**01_2i**	**100**	**100**	**100**	**100**	**100**	**100**	**100**	**100**	**100**	**NA**	**NA**	**NA**
6	02_2i	94.0	94.9	96.2	100	100	100	72.2	76.6	80.0	SOD	3	0
9	03_14i	100	100	100	100	100	100	100	100	100	NA	NA	NA
10	04_6i	94.4	94.9	96.2	100	100	100	68.8	73.5	80.0	FEN	2	1
**2.5**	**05_2i**	**100**	**100**	**100**	**100**	**100**	**100**	**100**	**100**	**100**	**NA**	**NA**	**NA**
**2.5**	**06_2i**	**100**	**100**	**100**	**100**	**100**	**100**	**100**	**100**	**100**	**NA**	**NA**	**NA**
5	07_4i	100	100	100	100	100	100	100	100	100	NA	NA	NA
**2.5**	**08_2i**	**100**	**100**	**100**	**100**	**100**	**100**	**100**	**100**	**100**	**NA**	**NA**	**NA**
7	09_10i	100	100	100	100	100	100	100	100	100	NA	NA	NA
8	10_12i	100	100	100	100	100	100	100	100	100	NA	NA	NA
Mean ± SEM (*n* = 10)		98.8 ± 0.77	99.0 ± 0.68	99.2 ± 0.51	100 ± 0.00	100 ± 0.00	100 ± 0.00	94.1 ± 3.94	95.0 ± 3.33	96.0 ± 2.67	NA	NA	NA

SVMs, support vector machines; model (e.g., 8i, 8 descriptors); you, Youden index; mcc, Matthews correlation coefficient; acc, accuracy; all, combined score for training and test sets; tra. training set, tes, test set; mis, misclassified drug or counterion; obs, observed class; pred, predicted class; classes: 0, antidepressant (red); 1, antipsychotic (blue); 2, anxiolytic (magenta); 3, counterion (gray). NA, not applicable. Statistical scores are expressed as percentages and defined in the Methods. Each model was started with a different random seed number and a training:test ratio of 21:5 compounds. Test set partition: stratified by CLASS using random selection. The three-letter code names for the drugs are given in Table 1. The four top-ranked models (shown in bold) used the following descriptors and relative sensitivities: 01_2i, F_AFRBWF (1.000), HBAch (0.946), random seed = 62402; 05_2i, FUnion (1.000), EEM_F6 (0.994), random seed = 61027; 06_2i, F_AFRBWF (1.000), HBAch (0.957), random seed = 24085; 08_21, FCation (1.000), Blbn_J (0.947), random seed = 2070. The members of the 4 test sets for the 4 tied first-place models were as follows: 01_2i: CIR, FLR, DRO, DIA, BRD; 05_21: ESC, FLS, BRO, MID, MAL; 06_2i: BUA, IMI, DRO, PRO, SOD; 08_21: BUA, FLS, BRO, FEN, MAL. Chemical descriptor definitions are listed in S1 Table. The rank for each model was determined by applying the RANK.AVG function in Microsoft Excel 365 to SUM(training metrics + test metrics + (100×$D_{min)}$ /D)), where D_{min} = minimum number of descriptors, and D = number of descriptors.
