# Supplementary material for: Distinguishing classes of neuroactive drugs based on computational physicochemical properties and experimental phenotypic profiling in planarians
Source: PLoS One. 2025 Jan 30;20(1):e0315394. doi: 10.1371/journal.pone.0315394 (PMC11781733; doi:10.1371/journal.pone.0315394)
Supplement: S9 Table — (PDF) [file pone.0315394.s019.pdf]

S9 Table. SVMs classification models using 3D molecular descriptors of drugs only.

**rank**	**model**	**you all**	**mcc all**	**acc all**	**you tra**	**mcc tra**	**acc tra**	**you tes**	**mcc tes**	**acc tes**	**mis**	**obs**	**pred**
**2**	**01_2i**	**100**	**100**	**100**	**100**	**100**	**100**	**100**	**100**	**100**	**NA**	**NA**	**NA**
7	02_4i	92.0	92.9	95.2	100	100	100	60.0	67.1	75.0	BUS	2	1
4.5	03_4i	100	100	100	100	100	100	100	100	100	NA	NA	NA
**2**	**04_2i**	**100**	**100**	**100**	**100**	**100**	**100**	**100**	**100**	**100**	**NA**	**NA**	**NA**
4.5	05_4i	100	100	100	100	100	100	100	100	100	NA	NA	NA
9	06_6i	92.0	92.9	95.2	100	100	100	50.0	64.5	75.0	OLA	1	0
**2**	**07_2i**	**100**	**100**	**100**	**100**	**100**	**100**	**100**	**100**	**100**	**NA**	**NA**	**NA**
6	08_6i	100	100	100	100	100	100	100	100	100	NA	NA	NA
8	09_4i	92.0	92.9	95.2	100	100	100	50.0	64.5	75.0	OLA	1	0
10	10_6i	92.0	92.9	95.2	100	100	100	50.0	57.7	75.0	PRO	1	0
Mean ± SEM (*n* = 10)		96.8 ± 1.30	97.2 ± 1.16	98.1 ± 0.78	100 ± 0.00	100 ± 0.00	100 ± 0.00	81.0 ± 7.81	85.4 ± 6.01	90.0 ± 4.08	NA	NA	NA

SVMs, support vector machines; model (e.g., 2i, 2 descriptors); you, Youden index; mcc, Matthews correlation coefficient; acc, accuracy; all, combined score for training and test sets; tra. training set, tes, test set; mis, misclassified drug or counterion; obs, observed class; pred, predicted class; classes: 0, antidepressant (red); 1, antipsychotic (blue); 2, anxiolytic (magenta). NA, not applicable. Statistical scores are expressed as percentages and defined in the Methods. Each model was started with a different random seed number and a training:test ratio of 17:4 compounds. Test set partition: stratified by CLASS using random selection. There were only 4 misclassifications: BUS, 2 x OLA, and PRO. The three-letter code names for the drugs are given in Table 1.
The three top-ranked models (shown in bold) used the following descriptors and relative sensitivities: 01_2i, FUnion (1.000), Bibn_J (0.966), random seed = 75745; 04_2i, M_POL (1.000), EEM_AFon (0.997), random seed = 26189; 07_2i, M_POL (1.000, NPA_AQon (0.998), random seed = 57410. Chemical descriptor definitions are listed in S1 Table. The rank for each model was determined by applying the RANK.AVG function in Microsoft Excel 365 to SUM(training metrics + test metrics + $(100\times D_{min)}/D)$), where D_{min} = minimum number of descriptors, and D = number of descriptors.
