# Supplementary material for: Distinguishing classes of neuroactive drugs based on computational physicochemical properties and experimental phenotypic profiling in planarians
Source: PLoS One. 2025 Jan 30;20(1):e0315394. doi: 10.1371/journal.pone.0315394 (PMC11781733; doi:10.1371/journal.pone.0315394)
Supplement: S10 Table — (PDF) [file pone.0315394.s020.pdf]

S10 Table. Calculated benchmark concentration values in logM.

Class	Chemical	Highest tested	CRO	STK	SHP	SCR	PTX	ANX	RSD	RSB	SPD	SB1	SB2	LBT	NSS
Antidepressant	BUP	-3.00	NaN	2.50	2.75	2.68	NaN	NaN	NaN	2.79	NaN	NaN	NaN	NaN	NaN
	CIT	-3.00	NaN	NaN	2.65	NaN	NaN	NaN	NaN	NaN	NaN	NaN	2.99	2.75	NaN
	DUL	-4.00	NaN	NaN	0.70	1.25	1.12	1.65	NaN	1.73	NaN	1.97	1.89	NaN	1.45
	ESC	-3.00	NaN	2.91	2.25	2.73	NaN	NaN	NaN	NaN	NaN	NaN	NaN	2.71	2.96
	FLU	-4.00	NaN	NaN	1.75	1.90	1.97	NaN	1.93	1.71	1.92	1.93	1.87	NaN	1.84
	IMI	-4.50	NaN	1.36	1.27	NaN	NaN	NaN	NaN	NaN	1.46	NaN	NaN	NaN	NaN
	SER	-4.00	NaN	NaN	1.64	0.95	NaN	NaN	NaN	NaN	NaN	NaN	NaN	NaN	NaN
Antipsychotic	ARI	-4.00	NaN	NaN	1.19	1.22	1.84	1.48	1.97	1.72	1.89	1.94	1.42	1.07	NaN
	BRO	-4.00	2.00	NaN	0.22	0.22	NaN	NaN	NaN	1.82	NaN	NaN	2.00	1.78	NaN
	CLO	-4.00	NaN	NaN	0.69	0.45	1.32	1.74	0.44	0.26	-0.05	0.44	-0.03	NaN	NaN
	DRO	-4.00	NaN	NaN	0.23	-0.22	0.42	1.65	0.91	0.39	0.39	0.46	0.36	1.67	1.82
	HAL	-4.00	NaN	NaN	1.53	1.93	NaN	NaN	NaN	1.91	1.93	1.97	1.93	1.80	NaN
	OLA	-3.50	2.00	NaN	-0.20	0.36	NaN	NaN	1.40	0.77	0.84	0.95	0.87	2.30	NaN
	PRO	-4.00	1.87	NaN	0.83	0.28	0.89	1.65	1.82	1.13	1.40	1.88	0.93	NaN	1.84
Anxiolytic	BUS	-4.00	NaN	NaN	NaN	1.45	1.74	NaN	NaN	1.69	NaN	1.96	1.88	NaN	NaN
	DIA	-4.00	NaN	NaN	1.30	1.90	NaN	1.93	NaN	1.82	1.91	1.97	1.89	1.68	NaN
	FEN	-3.25	NaN	NaN	NaN	NaN	NaN	NaN	NaN	NaN	NaN	NaN	NaN	NaN	NaN
	MID	-4.00	NaN	NaN	1.65	1.91	NaN	NaN	NaN	1.87	1.96	1.96	1.94	1.93	NaN
	TRA	-4.00	NaN	NaN	1.15	NaN	NaN	1.65	1.99	1.59	1.38	1.44	1.40	1.18	1.93
Counterion	OXA	-3.00	NaN	NaN	2.23	2.68	2.82	NaN	2.98	2.77	NaN	2.95	2.88	NaN	2.94
	NAB	-3.00	NaN	NaN	NaN	NaN	NaN	NaN	NaN	NaN	NaN	NaN	NaN	NaN	NaN

Endpoint abbreviations: CRO: crawl-out, STK: stickiness, SHP: body shape (any), SCR: scrunching, PTX: phototaxis, ANX: anxiety, RSD: resting_dark, RSB: resting_blue, SPD: speed_dark, SB1: speed_blue1, SB2: speed_blue2, LBT: locomotor bursts_total; NSS: noxious stimuli_strength. NaN indicates no BMC was determined and the chemical is inactive in that endpoint.
