# Supplementary material for: Distinguishing classes of neuroactive drugs based on computational physicochemical properties and experimental phenotypic profiling in planarians
Source: PLoS One. 2025 Jan 30;20(1):e0315394. doi: 10.1371/journal.pone.0315394 (PMC11781733; doi:10.1371/journal.pone.0315394)
Supplement: S11 Table — (PDF) [file pone.0315394.s021.pdf]

S11 Table. Converted BMC values in µM.

Class	Chemical	Highest tested	CRO	STK	SHP	SCR	PTX	ANX	RSD	RSB	SPD	SB1	SB2	LBT	NSS
Antidepressant	BUP	1000	NaN	316	562	478	NaN	NaN	NaN	621	NaN	NaN	NaN	NaN	NaN
	CIT	1000	NaN	NaN	446	NaN	NaN	NaN	NaN	NaN	NaN	NaN	9787	562	NaN
	DUL	100	NaN	NaN	5.01	17.6	13.2	44.7	NaN	53.3	NaN	93.5	77.2	NaN	28.3
	ESC	1000	NaN	814	178	537	NaN	NaN	NaN	NaN	NaN	NaN	NaN	513	922
	FLU	100	NaN	NaN	56.2	79.4	92.6	NaN	85.4	50.8	82. 7	85.8	74.5	NaN	69.0
	IMI	31.6	NaN	22.9	18.7	NaN	NaN	NaN	NaN	NaN	28.6	NaN	NaN	NaN	NaN
	SER	100	NaN	NaN	43.3	8.91	NaN	NaN	NaN	NaN	NaN	NaN	NaN	NaN	NaN
Antipsychotic	ARI	100	NaN	NaN	15.4	16.8	68.4	30.4	94.0	52.2	77.1	86.9	26.5	11.8	NaN
	BRO	100	100	NaN	1.65	1.68	NaN	NaN	NaN	65.9	NaN	NaN	100	60.5	NaN
	CLO	100	NaN	NaN	4.87	2.82	21.0	55.1	2.73	1.84	0.90	2.76	0.92	NaN	NaN
	DRO	100	NaN	NaN	1.70	0.60	2.63	44.7	8.18	2.44	2.44	2.91	2.30	47.0	66.6
	HAL	100	NaN	NaN	33.5	85.4	NaN	NaN	NaN	81.9	84.7	94.3	85.6	62.9	NaN
	OLA	316	100	NaN	0.63	2.29	NaN	NaN	25.0	5.95	6.86	9.01	7.41	199	NaN
	PRO	100	75	NaN	6.81	1.89	7.83	44.7	65.8	13.4	25.0	76.6	8.57	NaN	69.6
Anxiolytic	BUS	100	NaN	NaN	NaN	28.2	54.3	NaN	NaN	49.2	NaN	90.9	75.1	NaN	NaN
	DIA	100	NaN	NaN	19.9	79.4	NaN	84.2	NaN	66.2	80.8	94.4	78.2	47.5	NaN
	FEN	562	NaN	NaN	NaN	NaN	NaN	NaN	NaN	NaN	NaN	NaN	NaN	NaN	NaN
	MID	100	NaN	NaN	44.6	82.0	NaN	NaN	NaN	73.4	90.3	91.9	87.1	85.5	NaN
	TRA	100	NaN	NaN	14.1	NaN	NaN	44.7	97.3	39.2	23.9	27.8	25.1	15.1	84.8
Counterion	OXA	1000	NaN	NaN	170	478	665	NaN	963	592	NaN	891	751	NaN	864
	NAB	1000	NaN	NaN	NaN	NaN	NaN	NaN	NaN	NaN	NaN	NaN	NaN	NaN	NaN

Endpoint abbreviations: CRO: crawl-out, STK: stickiness, SHP: body shape (any), SCR: scrunching, PTX: phototaxis, ANX: anxiety, RSD: resting_dark, RSB: resting_blue, SPD: speed_dark, SB1: speed_blue1, SB2: speed_blue2, LBT: locomotor bursts_total; NSS: noxious stimuli_strength. NaN indicates no BMC was determined and the chemical is inactive in that endpoint.
