# Supplementary material for: Distinguishing classes of neuroactive drugs based on computational physicochemical properties and experimental phenotypic profiling in planarians
Source: PLoS One. 2025 Jan 30;20(1):e0315394. doi: 10.1371/journal.pone.0315394 (PMC11781733; doi:10.1371/journal.pone.0315394)
Supplement: S12 Table — (PDF) [file pone.0315394.s022.pdf]

S12 Table. Median normalized responses for each endpoint after exposure to the counterions.

Condition[a]	CRO	STK	SHP	SHP1	SHP2	SHP3	SHP4	SHP5	SCR	PTX	ANX	RSD	RSB	SPD	SB1	SB2	LBT	NSS
MAL_200	0	13	0	0	0	0	0	0	**33**	0	24	-13	-5	-20	-33	-24	-2	-39
OXA_100	0	0	13	0	0	0	0	13	8	0	25	-2	2	-7	-25	-8	7	-12
OXA_316	0	**38**	**63**	0	0	0	0	**58**	0	8	-25	33	23	-42	-63	-69	**32**	**-68**
OXA_1000	0	0	**63**	**50**	**42**	0	0	0	**83**	**54**	-19	**61**	**64**	-53	**-88**	**-90**	4	**-57**
SOB_100	0	0	0	0	0	0	0	0	4	0	-11	0	0	-11	-10	-3	1	-5
SOB_300	0	0	0	0	0	0	0	0	4	0	12	0	0	2	-8	-11	0	17
SOB_1000	0	0	0	0	0	0	0	0	0	0	13	-1	0	15	34	18	-6	20
SOC_3470	4	0	0	0	0	0	0	0	13	0	-4	3	-9	-7	19	-3	1	-5

Bold values indicate significant responses outside of the range of the respective benchmark response for that endpoint. Endpoint abbreviations: CRO: crawl-out, STK: stickiness, SHP: body shape (numbers refer to distinct body shape classes. 1: contracted, 2: C-shape, 3: Corkscrew, 4: Pharynx extrusion, 5: Hyperkinesis), SCR: scrunching, PTX: phototaxis, ANX: anxiety, RSD: resting_dark, RSB: resting_blue, SPD: speed_dark, SB1: speed_blue1, SB2: speed_blue2, LBT: locomotor bursts_total; NSS: noxious stimuli_strength.

[a]Condition refers to the chemical (3 digit code) followed by the concentration (μM). OXA: oxalic acid, MAL: maleic acid, SOC: sodium chloride, SOB: sodium bromide.
