# Supplementary material for: Distinguishing classes of neuroactive drugs based on computational physicochemical properties and experimental phenotypic profiling in planarians
Source: PLoS One. 2025 Jan 30;20(1):e0315394. doi: 10.1371/journal.pone.0315394 (PMC11781733; doi:10.1371/journal.pone.0315394)
Supplement: S13 Table — (PDF) [file pone.0315394.s023.pdf]

S13 Table. pH at highest tested concentration.

**Common name**	**Abbreviation**	**Highest tested nominal concentration** (µM)	**pH**[a]	**pH w/ probe**[b]
Aripiprazole HCl	ARI	100	8	6.49
L-Ascorbic acid	--	100	6.5	4.41
Bromperidol	BRO	100	8	6.97
Bupropion HCl	BUP	1000	7	5.69
Buspirone HCl	BUS	100	7	6.27
Citalopram HBr	CIT	1000	7	6.42
Clozapine	CLO	100	8	7.71
Diazepam	DIA	100	7	6.26
Droperidol	DRO	100	8	7.54
Duloxetine HCl	DUL	100	8	6.55
Escitalopram oxalate*	ESC	1000	6	3.63
Fenobam	FEN	562	7	6.03
Fluoxetine HCl	FLU	100	7.5	6.17
Haloperidol	HAL	100	7.5	7.33
Imipramine HCl	IMI	31.6	7.5	6.68
Maleic acid	MAL	200	6	3.84
Midazolam	MID	100	7	5.73
Olanzapine	OLA	316	9	8.57
Oxalic acid**	OXA	1000	5	3.06
Prochlorperazine dimaleate	PRO	100	6	4.42
Sertraline HCl	SER	100	7.5	5.64
Sodium bromide	SOB	1000	7	6.20
Sodium chloride	SOC	3470	7	6.33
D-Sorbitol	--	100	8	6.07
Tracazolate HCl	TRA	100	6.5	4.72

pH of 0.5% DMSO = 6.63 ± 0.17, IO water = 6.72 ± 0.41 (mean ± standard deviation, from 5 measurements). To determine whether pH was the reason we saw effects at these concentrations, we also measured 100 µM and 316 µM with the pH probe and obtained *for escilatopram 4.58 and 3.86, respectively, and ** for oxalic acid 4.23 and 3.54 respectively.

[a]pH measured using a pH-strip (VWR, Radnor, PA)

[b] pH measured with Apera PH60-MS pH Tester kit (Apera Instruments, Columbus, Ohio)
