# Supplementary material for: Distinguishing classes of neuroactive drugs based on computational physicochemical properties and experimental phenotypic profiling in planarians
Source: PLoS One. 2025 Jan 30;20(1):e0315394. doi: 10.1371/journal.pone.0315394 (PMC11781733; doi:10.1371/journal.pone.0315394)
Supplement: S14 Table — (PDF) [file pone.0315394.s024.pdf]

S14 Table. Median normalized responses for extreme pHs.

pH	CRO	STK	SHP	SCR	PTX	ANX	RSD	RSB	SPD	SB1	SB2	LBT	NSS
3	17	**33**	**95**	**70**	35	32	**93**	**97**	**-81**	**-98**	**-98**	10	**-73**
3.5	0	22	**83**	**63**	33	32	**92**	**96**	**-82**	**-98**	**-97**	14	**-66**
4	0	4	4	17	4	27	8	5	-38	-46	-29	6	-45
5	0	0	0	4	0	-12	2	0	7	18	50	-1	-40
6	0	0	0	8	0	-4	-1	-1	16	20	101	-4	-14
9	0	0	0	0	0	-12	0	-8	5	5	78	-4	4

Bold values indicate significant responses outside of the range of the respective benchmark response for that endpoint. Endpoint abbreviations: CRO: crawl-out, STK: stickiness, SHP: body shape, SCR: scrunching, PTX: phototaxis, ANX: anxiety, RSD: resting_dark, RSB: resting_blue, SPD: speed_dark, SB1: speed_blue1, SB2: speed_blue2, LBT: locomotor bursts_total; NSS: noxious stimuli_strength.
