# Supplementary material for: Distinguishing classes of neuroactive drugs based on computational physicochemical properties and experimental phenotypic profiling in planarians
Source: PLoS One. 2025 Jan 30;20(1):e0315394. doi: 10.1371/journal.pone.0315394 (PMC11781733; doi:10.1371/journal.pone.0315394)
Supplement: S15 Table — (PDF) [file pone.0315394.s025.pdf]

S15 Table. ANNE classification models using behavioral responses to 18 drugs (-FEN) and 4 counterions.

rank	model	you all	mcc all	acc all	you tra	mcc tra	acc tra	you tes	mcc tes	acc tes	mis	obs	pred
**1**	**01_1n7**	**87.6**	**87.8**	**90.9**	**91.6**	**92.5**	**94.1**	**81.3**	**76.6**	**80.0**	**DIA**	**2**	**1**
											**DUL**	**0**	**2**
6	02_1n6	79.7	82.8	86.4	90.5	92.3	94.1	50.0	56.7	60.0	ARI	1	0
											DIA	2	0
											MID	2	0
10	03_1n5	68.1	70.1	77.3	84.1	84.5	88.2	31.3	33.4	40.0	CIT	0	3
											DIA	2	1
											IMI	0	2
											OXA	3	1
											TRA	2	1
8	04_2n1	68.9	69.9	77.3	76.2	76.5	82.4	43.8	46.8	60.0	BRO	1	2
											DUL	0	1
											FLU	0	1
											MID	2	1
											OXA	3	2
5	05_1n3	79.7	82.8	86.4	90.5	92.3	94.1	50.0	56.7	60.0	BRO	1	2
											DUL	0	1
											FLU	0	1
7	06_1n6	72.6	76.1	81.8	81.4	84.3	88.2	50.0	56.7	60.0	BUP	0	1
											BUS	2	1
											DIA	2	0
											OXA	3	1
2	07_1n5	89.3	88.8	90.9	100	100	100	50.0	57.7	60.0	BUP	0	3
											IMI	0	3
3	08_1n4	87.6	87.8	90.9	100	100	100	50.0	50.0	60.0	ARI	1	2
											MID	2	0
9	09_1n3	69.8	69.8	77.3	85.2	84.4	88.2	27.8	31.5	40.0	ARI	1	0
											DIA	2	1
											DUL	0	1
											IMI	0	2
											SER	0	2
4	10_1n5	75.1	76.0	81.8	78.3	78.3	82.4	71.4	71.4	80.0	BUP	0	1
											DIA	2	1
											IMI	0	3
											OXA	3	2
Mean ± SEM (*n* = 10)		77.8 ± 2.59	79.2 ± 2.46	84.1 ± 1.82	87.8 ± 2.61	88.5 ± 2.60	91.2 ± 2.01	50.6 ± 5.07	53.8 ± 4.53	60.0 ± 4.22	NA	NA	NA

ANNE, artificial neural network ensemble; model (e.g., 1n7, 1 neuron and 7 variables); you, Youden index; mcc, Matthews correlation coefficient; acc, accuracy; all, combined score for training and test sets; tra, training set; tes, test set; mis, misclassified drug; obs, observed class; pred, predicted class; classes: 0, antidepressant; 1, antipsychotic; 2, anxiolytic; 3, counterion. NA, not applicable. Statistical scores are expressed as percentages and defined in the Methods. Each model was started with a different random seed number and a training:test ratio of 17:5 compounds. Test set partition: stratified by CLASS using random selection. Color codes: red, antidepressant; blue, antipsychotic; magenta, anxiolytic; gray, counterion. The three-letter code names for the drugs are given in Table 1. The top-ranked model (shown in bold) used the following descriptors and relative sensitivities: SHPH_12 (1.000), NSS_08 (0.996), SB2_09 (0.993), SCR_09 (0.941), PTX_10 (0.753), RSD_11 (0.600), and LBT_08 (0.597), random seed = 45418. Behavioral descriptor definitions are given in S7 Fig and Tables 2 and 3. The rank for each model was determined by applying the RANK.AVG function in Microsoft Excel 365 to SUM(training metrics + test metrics + (100×N_{min}/N) + (100×D_{min} /D)), where N_{min} = minimum number of neurons, N = number of neurons, D_{min} = minimum number of descriptors, and D = number of descriptors.
