# Supplementary material for: Distinguishing classes of neuroactive drugs based on computational physicochemical properties and experimental phenotypic profiling in planarians
Source: PLoS One. 2025 Jan 30;20(1):e0315394. doi: 10.1371/journal.pone.0315394 (PMC11781733; doi:10.1371/journal.pone.0315394)
Supplement: S16 Table — (PDF) [file pone.0315394.s026.pdf]

**S16 Table. ANNE classification models using behavioral responses to 18 drugs (-FEN).**

| rank | model | you all | mcc all | acc all | you tra | mcc tra | acc tra | you tes | mcc tes | acc tes | mis | obs | pred |
|---|---|---|---|---|---|---|---|---|---|---|---|---|---|
| 6 | 01_1n4 | 82.9 | 83.3 | 88.9 | 100 | 100 | 100 | 30.0 | 30.0 | 50.0 | BUS<br>DUL | 2<br>0 | 1<br>2 |
| 2 | 02_1n2 | 80.0 | 83.7 | 88.9 | 87.3 | 89.5 | 92.9 | 60.0 | 67.1 | 75.0 | MID<br>TRA | 2<br>2 | 0<br>1 |
| 5 | 03_1n3 | 82.9 | 84.5 | 88.9 | 100 | 100 | 100 | 40.0 | 51.6 | 50.0 | DUL<br>FLU | 0<br>0 | 1<br>1 |
| 9 | 04_1n4 | 72.9 | 74.3 | 83.3 | 89.7 | 89.7 | 92.9 | 30.0 | 38.7 | 50.0 | ARI<br>CIT<br>MID | 1<br>0<br>2 | 0<br>1<br>1 |
| 7 | 05_1n5 | 81.4 | 83.9 | 88.9 | 100 | 100 | 100 | 30.0 | 39.0 | 50.0 | DUL<br>TRA | 0<br>2 | 1<br>1 |
| **1** | **06_1n4** | **92.9** | **92.0** | **94.4** | **91.3** | **89.9** | **92.9** | **100** | **100** | **100** | **ARI** | **1** | **2** |
| 8 | 07_1n5 | 81.4 | 83.9 | 88.9 | 100 | 100 | 100 | 30.0 | 38.7 | 50.0 | BUS<br>DUL | 2<br>0 | 1<br>1 |
| 4 | 08_1n4 | 85.7 | 84.5 | 88.9 | 90.5 | 89.8 | 92.9 | 60.0 | 67.1 | 75.0 | ARI<br>DUL | 1<br>0 | 2<br>2 |
| 10 | 09_1n4 | 75.7 | 75.0 | 83.3 | 91.3 | 89.9 | 92.9 | 30.0 | 30.0 | 50.0 | HAL<br>DUL<br>TRA | 1<br>0<br>2 | 2<br>2<br>1 |
| 3 | 10_1n5 | 90.0 | 91.8 | 94.4 | 100 | 100 | 100 | 60.0 | 67.1 | 75.0 | DIA | 2 | 1 |
| Mean ± SEM (*n* = 10) | | 82.6 ± 1.89 | 83.7 ± 1.83 | 88.9 ± 1.17 | 95.0 ± 1.70 | 94.9 ± 1.71 | 96.4 ± 1.18 | 47.0 ± 7.31 | 52.9 ± 7.04 | 62.5 ± 5.59 | NA | NA | NA |

ANNE, artificial neural network ensemble; model (e.g., 1n4, 1 neuron and 4 variables); you, Youden index; mcc, Matthews correlation coefficient; acc, accuracy; all, combined score for training and test sets; tra, training set; tes, test set; mis, misclassified drug; obs, observed class; pred, predicted class; classes: 0, antidepressant; 1, antipsychotic; 2, anxiolytic. NA, not applicable. Statistical scores are expressed as percentages and defined in the Methods. Each model was started with a different random seed number and a training:test ratio of 14:4 compounds. Test set partition: stratified by CLASS using random selection. Color codes: red, antidepressant; blue, antipsychotic; magenta, anxiolytic. The three-letter code names for the drugs are given in Table 1. The top-ranked model (shown in bold) used the following behavioral descriptors and relative sensitivities: SPD_09 (1.000), ANX_09 (0.999), SHP_08 (0.997), SB2_09 (0.992), random seed = 2887. Behavioral descriptor definitions are given in S7 Fig and Tables 2 and 3. The rank for each model was determined by applying the RANK.AVG function in Microsoft Excel 365 to SUM(training metrics + test metrics + $(100 \times N_{min}/N) + (100 \times D_{min})/D)$), where $N_{min}$ = minimum number of neurons, $N$ = number of neurons, $D_{min}$ = minimum number of descriptors, and $D$ = number of descriptors.
