# Supplementary material for: Distinguishing classes of neuroactive drugs based on computational physicochemical properties and experimental phenotypic profiling in planarians
Source: PLoS One. 2025 Jan 30;20(1):e0315394. doi: 10.1371/journal.pone.0315394 (PMC11781733; doi:10.1371/journal.pone.0315394)
Supplement: S17 Table — (PDF) [file pone.0315394.s027.pdf]

S17 Table. SVMs classification models using behavioral responses to 18 drugs (-FEN) and 4 counterions.

rank	model	you all	mcc all	acc all	you tra	mcc tra	acc tra	you tes	mcc tes	acc tes	mis	obs	pred
4	01_6i	93.8	94.1	95.5	100	100	100	72.2	76.6	80.0	DIA	2	3
3	02_4i	92.9	94.0	95.5	100	100	100	66.7	75.6	80.0	MID	2	0
6	03_12i	92.9	94.0	95.5	100	100	100	72.2	76.6	80.0	BUS	2	1
5	04_12i	93.8	94.1	95.5	100	100	100	78.6	73.5	80.0	ARI	1	0
9	05_4i	88.4	87.9	90.9	100	100	100	50.0	50.0	60.0	ARI<br>BUS	1<br>2	2<br>3
**1**	**06_10i**	**100**	**100**	**100**	**100**	**100**	**100**	**100**	**100**	**100**	**NA**	**NA**	**NA**
10	07_8i	88.4	87.9	90.9	100	100	100	50.0	50.0	60.0	DIA<br>IMI	2<br>0	3<br>2
8	08_4i	86.7	88.2	90.9	91.9	92.3	84.1	66.7	75.6	80.0	BUS<br>MID	2<br>2	3<br>0
2	09_12i	100	100	100	100	100	100	100	100	100	NA	NA	NA
7	10_2i	86.7	88.2	90.9	100	100	100	33.3	50.0	60.0	HAL<br>OXA	1<br>3	0<br>0
Mean ± SEM (*n* = 10)		92.4 ± 1.55	92.8 ± 1.49	94.6 ± 1.14	99.2 ± 0.81	99.2 ± 9.77	98.4 ± 1.59	69.0 ± 6.70	72.8 ± 5.85	78.0 ± 4.67	NA	NA	NA

SVMs, support vector machines; model (e.g., 6i, 6 variables); you, Youden index; mcc, Matthews correlation coefficient; acc, accuracy; all, combined score for training and test sets; tra, training set; tes, test set; mis, misclassified drug; obs, observed class; pred, predicted class; classes: 0, antidepressant; 1, antipsychotic; 2, anxiolytic; 3, counterion. NA, not applicable. Statistical scores are expressed as percentages and defined in the Methods. Each model was started with a different random seed number and a training:test ratio of 17:5 compounds. Test set partition: stratified by CLASS using random selection. Color codes: red, antidepressant; blue, antipsychotic; magenta, anxiolytic; gray, counterion. The three-letter code names for the drugs are given in Table 1. Behavioral descriptor definitions are given in S7 Fig and Tables 2 and 3. The top-ranked model (shown in bold) used the following behavioral descriptors and relative sensitivities: NSS_08 (1.000), PTX_08 (0.907), SHPH_12 (0.743), SB2_09 (0.702), SUI_09 (0.679), SCR_09 (0.676), SHP_10 (0.661), RSD_08 (0.652), SHP6_12 (0.649), and SHP3_10 (0.629), random seed = 82051. The rank for each model was determined by applying the RANK.AVG function in Microsoft Excel 365 to SUM(training metrics + test metrics + (100×$D_{min)}$ /D)), where D_{min} = minimum number of descriptors, and D = number of descriptors.
