# Supplementary material for: Distinguishing classes of neuroactive drugs based on computational physicochemical properties and experimental phenotypic profiling in planarians
Source: PLoS One. 2025 Jan 30;20(1):e0315394. doi: 10.1371/journal.pone.0315394 (PMC11781733; doi:10.1371/journal.pone.0315394)
Supplement: S18 Table — (PDF) [file pone.0315394.s028.pdf]

S18 Table. SVMs classification models using behavioral responses to 18 drugs (-FEN).

rank	model	you all	mcc all	acc all	you tra	mcc tra	acc tra	you tes	mcc tes	acc tes	mis	obs	pred
7	01_5i	92.9	92.0	94.4	100	100	100	75.0	67.1	75.0	FLU	0	2
10	02_9i	90.0	91.8	94.4	100	100	100	60.0	67.1	75.0	BUS	2	1
8.5	03_7i	90.0	91.8	94.4	100	100	100	60.0	67.1	75.0	BUS	2	1
5	04_2i	91.4	91.9	94.4	100	100	100	70.0	75.0	75.0	DUL	0	1
3	05_7i	100	100	100	100	100	100	100	100	100	NA	NA	NA
6	06_4i	91.4	91.9	94.4	100	100	100	70.0	70.0	75.0	IMI	0	1
**1**	**07_3i**	**100**	**100**	**100**	**100**	**100**	**100**	**100**	**100**	**100**	**NA**	**NA**	**NA**
2	08_5i	100	100	100	100	100	100	100	100	100	NA	NA	NA
8.5	09_7i	90.0	91.8	94.4	100	100	100	60.0	67.1	75.0	BUS	2	1
4	10_9i	100	100	100	100	100	100	100	100	100	NA	NA	NA
Mean ± SEM (*n* = 10)		94.6 ± 1.50	95.1 ± 1.33	96.6 ± 0.91	100 ± 0.00	100 ± 0.00	100 ± 0.00	79.5 ± 5.80	81.3 ± 5.13	85.0 ± 4.08	NA	NA	NA

SVMs, support vector machines; model (e.g., 5i, 6 variables); you, Youden index; mcc, Matthews correlation coefficient; acc, accuracy; all, combined score for training and test sets; tra, training set; tes, test set; mis, misclassified drug; obs, observed class; pred, predicted class; classes: 0, antidepressant; 1, antipsychotic; 2, anxiolytic. NA, not applicable. Statistical scores are expressed as percentages and defined in the Methods. Each model was started with a different random seed number and a training:test ratio of 14:4 compounds. Test set partition: stratified by CLASS using random selection. Color codes: red, antidepressant; blue, antipsychotic; magenta, anxiolytic. The three-letter code names for the drugs are given in Table 1. The three-letter code names for the drugs are given in Table 1. Behavioral descriptor definitions are given in S7 Fig and Tables 2 and 3. The top-ranked model (shown in bold) used the following behavioral descriptors and relative sensitivities:, random seed = NSS_08 (1.000), RSD_10 (0.888), SB2_12 (0.888), random seed = 3034. The rank for each model was determined by applying the RANK.AVG function in Microsoft Excel 365 to SUM(training metrics + test metrics + (100×$D_{min)}$ /*D*)), where D_{min} = minimum number of descriptors, and *D* = number of descriptors.
