# Supplementary material for: Distinguishing classes of neuroactive drugs based on computational physicochemical properties and experimental phenotypic profiling in planarians
Source: PLoS One. 2025 Jan 30;20(1):e0315394. doi: 10.1371/journal.pone.0315394 (PMC11781733; doi:10.1371/journal.pone.0315394)
Supplement: S19 Table — (PDF) [file pone.0315394.s029.pdf]

S19 Table. ANNE classification models using behavioral responses to 19 drugs (+FEN) and 4 counterions.

rank	model	you all	mcc all	acc all	you tra	mcc tra	acc tra	you tes	mcc tes	acc tes	mis	obs	pred
2	01_1n8	82.3	83.8	87.0	79.2	80.2	83.3	100	100	100	BUS	2	0
											FEN	2	3
											MID	2	3
8	02_1n6	89.2	89.0	91.3	100	100	100	50.0	56.7	60.0	FEN	2	3
											IMI	0	3
10	03_1n6	70.0	70.9	78.3	77.7	79.8	83.3	50.0	50.0	60.0	ARI	1	2
											BUP	0	1
											BUS	2	1
											FEN	2	0
											IMI	0	1
6	04_1n8	81.3	83.0	87.0	82.8	85.7	88.9	72.2	76.6	80.0	FEN	2	3
											MID	2	0
											OXA	3	0
**1**	**05_1n8**	**87.7**	**88.4**	**91.3**	**84.5**	**85.2**	**88.9**	**100**	**100**	**100**	**FEN**	**2**	**3**
											**OXA**	**3**	**0**
4	06_1n8	87.9	88.9	91.3	92.9	92.9	94.4	72.2	76.6	80.0	FEN	2	3
											MID	2	0
7	07_1n2	56.7	61.4	69.6	54.2	59.4	66.7	72.2	76.6	80.0	BRO	1	0
											DIA	2	1
											DRO	1	0
											FEN	2	1
											MID	2	0
											OLA	1	0
											TRA	2	0
3	08_1n5	82.3	82.3	87.0	85.0	85.4	88.9	81.3	76.6	80.0	ARI	1	0
											DIA	2	1
											IMI	0	2
5	09_1n5	83.1	82.7	87.0	85.0	85.0	88.9	75.0	75.0	80.0	BUS	2	1
											DUL	0	3
											HAL	1	2
9	10_1n4	76.9	77.9	82.6	84.9	86.0	88.9	50.0	50.0	60.0	BUS	2	1
											CIT	0	2
											DUL	0	1
											IMI	0	2
Mean ± SEM (n = 10)		79.7 ± 3.13	80.8 ± 2.78	85.2 ± 2.17	82.6 ± 3.76	84.0 ± 3.31	87.2 ± 2.75	72.3 ± 5.88	73.8 ± 5.61	78.0 ± 4.67	NA	NA	NA

ANNE, artificial neural network ensemble; model (e.g., 1n8, 1 neuron and 8 variables); you, Youden index; mcc, Matthews correlation coefficient; acc, accuracy; all, combined score for training and test sets; tra, training set; tes, test set; mis, misclassified drug or counterion; obs, observed class; pred, predicted class; classes: 0, antidepressant; 1, antipsychotic; 2, anxiolytic; 3, counterion. NA, not applicable. Statistical scores are expressed as percentages and defined in the Methods. Each model was started with a different random seed number and a training:test ratio of 18:5 compounds. Test set partition: stratified by CLASS using random selection. Color codes: red, antidepressant; blue, antipsychotic; magenta, anxiolytic; gray, counterion. The three-letter code names for the drugs are given in Table 1. The top-ranked model (shown in bold) used the following behavioral descriptors and relative sensitivities: STK_08 (1.000), SUI_10 (0.987), SPD_12 (0.987), SHP4_10 (0.982), SHP2_11 (0.976), SUI_12 (0.974), SHP4_11 (0.969), RSB_10 (0.968), random seed = 7142. Behavioral descriptor definitions are given in S7 Fig and Tables 2 and 3. The rank for each model was determined by applying the RANK.AVG function in Microsoft Excel 365 to SUM(training metrics + test metrics + ($100 \times N_{min}/N$) + ($100 \times D_{min)}$ /D)), where N_{min} = minimum number of neurons, N = number of neurons, D_{min} = minimum number of descriptors, and D = number of descriptors.
