# Supplementary material for: Distinguishing classes of neuroactive drugs based on computational physicochemical properties and experimental phenotypic profiling in planarians
Source: PLoS One. 2025 Jan 30;20(1):e0315394. doi: 10.1371/journal.pone.0315394 (PMC11781733; doi:10.1371/journal.pone.0315394)
Supplement: S20 Table — (PDF) [file pone.0315394.s030.pdf]

S20 Table. ANNE classification models using behavioral responses to 19 drugs (+FEN).

rank	model	you all	mcc all	acc all	you tra	mcc tra	acc tra	you tes	mcc tes	acc tes	mis	obs	pred
7	01_1n7	74.4	77.0	84.2	77.7	81.1	86.7	70.0	70.0	75.0	BUP	0	1
											BUS	2	1
											DIA	2	0
8	02_1n3	68.1	69.2	78.9	79.7	79.7	86.7	40.0	51.6	50.0	ARI	1	0
											HAL	1	2
											PRO	1	0
											TRA	2	1
6	03_2n1	69.7	69.5	78.9	73.0	74.0	80.0	60.0	67.1	75.0	FLU	0	2
											HAL	1	2
											IMI	0	2
											MID	2	0
5	04_1n6	77.7	78.7	84.2	79.7	81.4	86.7	70.0	70.0	75.0	BUP	0	1
											CIT	0	2
											IMI	0	2
4	05_1n4	83.2	84.3	89.5	90.5	90.5	93.3	60.0	67.1	75.0	ARI	1	0
											DIA	2	1
**1**	**06_1n7**	**82.4**	**85.3**	**89.5**	**78.4**	**81.8**	**86.7**	**100**	**100**	**100**	**DIA**	**2**	**0**
											**TRA**	**2**	**0**
9	07_1n7	83.2	84.3	89.5	100	100	100	20.0	22.4	50.0	ARI	1	0
											TRA	2	1
3	08_1n7	75.2	76.8	84.2	69.6	71.0	80.0	100	100	100	ARI	1	0
											HAL	1	0
											MID	2	1
10	09_1n5	75.2	76.8	84.2	100	100	100	-10.0	-12.9	25.0	ARI	1	0
											MID	2	1
											PRO	1	0
2	10_2n1	68.9	69.2	78.9	61.5	61.5	73.3	100	100	100	ARI	1	0
											DUL	0	1
											HAL	1	2
											PRO	1	0
Mean ± SEM (*n* = 10)		75.8 ± 1.84	77.1 ± 1.99	84.2 ± 1.37	81.0 ± 3.95	82.1 ± 3.85	87.3 ± 2.71	61.0 ± 11.5	63.5 ± 11.4	72.5 ± 7.86	NA	NA	NA

ANNE, artificial neural network ensemble; model (e.g., 1n7, 1 neuron and 7 variables); you, Youden index; mcc, Matthews correlation coefficient; acc, accuracy; all, combined score for training and test sets; tra, training set; tes, test set; mis, misclassified drug; obs, observed class; pred, predicted class; classes: 0, antidepressant; 1, antipsychotic; 2, anxiolytic. NA, not applicable. Statistical scores are expressed as percentages and defined in the Methods. Each model was started with a different random seed number and a training:test ratio of 15:4 compounds. Test set partition: stratified by CLASS using random selection. Color codes: red, antidepressant; blue, antipsychotic; magenta, anxiolytic. The three-letter code names for the drugs are given in Table 1. The top-ranked model (shown in bold) used the following behavioral descriptors and relative sensitivities: SPD_10 (1.000), RSB_10 (0.927), SHP_08 (0.912), SCR_08 (0.701), NSS_12 (0.668), SHPH_09 (0.664), SB2_09 (0.655), random seed = 12676. Behavioral descriptor definitions are given in S7 Fig and Tables 2 and 3. The rank for each model was determined by applying the RANK.AVG function in Microsoft Excel 365 to SUM(training metrics + test metrics + ($100 \times N_{min}/N$) + ($100 \times D_{min}/D$)), where N_{min} = minimum number of neurons, N = number of neurons, D_{min} = minimum number of descriptors, and D = number of descriptors.
