# Supplementary material for: Distinguishing classes of neuroactive drugs based on computational physicochemical properties and experimental phenotypic profiling in planarians
Source: PLoS One. 2025 Jan 30;20(1):e0315394. doi: 10.1371/journal.pone.0315394 (PMC11781733; doi:10.1371/journal.pone.0315394)
Supplement: S21 Table — (PDF) [file pone.0315394.s031.pdf]

**S21 Table. SVMs classification models using behavioral responses to 19 drugs (+FEN) and 4 counterions.**

| rank | model | you all | mcc all | acc all | you tra | mcc tra | acc tra | you tes | mcc tes | acc tes | mis | obs | pred |
|---|---|---|---|---|---|---|---|---|---|---|---|---|---|
| 6 | 01_10i | 93.3 | 94.3 | 95.7 | 100 | 100 | 100 | 64.3 | 69.4 | 80.0 | OXA | 3 | 0 |
| 4.5 | 02_8i | 94.6 | 94.4 | 95.7 | 100 | 100 | 100 | 68.8 | 73.5 | 80.0 | IMI | 0 | 2 |
| 2 | 03_10i | 94.9 | 94.4 | 95.7 | 100 | 100 | 100 | 77.8 | 77.8 | 80.0 | FLU | 0 | 3 |
| 8 | 04_12i | 89.2 | 89.2 | 91.3 | 100 | 100 | 100 | 55.6 | 63.0 | 60.0 | BUP | 0 | 2 |
| | | | | | | | | | | | IMI | 0 | 2 |
| 10 | 05_12i | 87.9 | 88.4 | 91.3 | 100 | 100 | 100 | 44.4 | 47.1 | 60.0 | FLU | 0 | 2 |
| | | | | | | | | | | | OXA | 3 | 0 |
| **1** | **06_4i** | **93.6** | **94.3** | **95.6** | **100** | **100** | **100** | **66.7** | **75.6** | **80.0** | **MID** | **2** | **0** |
| 4.5 | 07_8i | 93.6 | 94.3 | 95.7 | 100 | 100 | 100 | 66.7 | 75.6 | 80.0 | MID | 2 | 0 |
| 3 | 08_2i | 87.4 | 88.8 | 91.3 | 100 | 100 | 100 | 42.9 | 46.3 | 60.0 | ARI | 1 | 0 |
| | | | | | | | | | | | OXA | 3 | 0 |
| 7 | 09_6i | 88.7 | 88.9 | 91.3 | 100 | 100 | 100 | 55.6 | 58.9 | 60.0 | BRO | 1 | 0 |
| | | | | | | | | | | | HAL | 1 | 2 |
| 9 | 10_14i | 88.5 | 88.9 | 91.3 | 100 | 100 | 100 | 57.1 | 53.5 | 60.0 | ARI | 1 | 2 |
| | | | | | | | | | | | OXA | 3 | 2 |
| Mean ± SEM (*n* = 10) | | 91.2 ± 0.97 | 91.6 ± 0.92 | 93.5 ± 0.73 | 100 ± 0.00 | 100 ± 0.00 | 100 ± 0.00 | 60.0 ± 3.47 | 64.1 ± 3.82 | 70.0 ± 3.33 | NA | NA | NA |

SVMs, support vector machines; model (e.g., 10i, 10 variables); you, Youden index; mcc, Matthews correlation coefficient; acc, accuracy; all, combined score for training and test sets; tra, training set; tes, test set; mis, misclassified drug; obs, observed class; pred, predicted class; classes: 0, antidepressant; 1, antipsychotic; 2, anxiolytic; 3, counterion. NA, not applicable. Statistical scores are expressed as percentages and defined in the Methods. Each model was started with a different random seed number and a training:test ratio of 18:5 compounds. Test set partition: stratified by CLASS using random selection. Color codes: red, antidepressant; blue, antipsychotic; magenta, anxiolytic; gray, counterion. The three-letter code names for the drugs are given in Table 1. The top-ranked model (shown in bold) used the following behavioral descriptors and relative sensitivities: NSS_08 (1.000), SHPH_12 (0.756), PTX_08 (0.737), ANX_09 (0.731), random seed = 38336. Behavioral descriptor definitions are given in S7 Fig and Tables 2 and 3. The rank for each model was determined by applying the RANK.AVG function in Microsoft Excel 365 to SUM(training metrics + test metrics + (100×$D_{min)}$ /$D$)), where $D_{min}$ = minimum number of descriptors, and $D$ = number of descriptors.
