# Supplementary material for: Distinguishing classes of neuroactive drugs based on computational physicochemical properties and experimental phenotypic profiling in planarians
Source: PLoS One. 2025 Jan 30;20(1):e0315394. doi: 10.1371/journal.pone.0315394 (PMC11781733; doi:10.1371/journal.pone.0315394)
Supplement: S22 Table — (PDF) [file pone.0315394.s032.pdf]

S22 Table. SVMs classification models using behavioral responses to 19 drugs (+FEN).

**rank**	**model**	**you all**	**mcc all**	**acc all**	**you tra**	**mcc tra**	**acc tra**	**you tes**	**mcc tes**	**acc tes**	**mis**	**obs**	**pred**
2.5	01_4i	100	100	100	100	100	100	100	100	100	NA	NA	NA
5.5	02_2i	92.0	92.4	94.7	100	100	100	70.0	70.0	75.0	BUP	0	1
**1**	**03_3i**	**100**	**100**	**100**	**100**	**100**	**100**	**100**	**100**	**100**	**NA**	**NA**	**NA**
2.5	04_4i	100	100	100	100	100	100	100	100	100	NA	NA	NA
4	05_2i	84.9	84.5	89.5	81.1	80.5	86.7	100	100	100	DUL<br>HAL	0<br>1	2<br>0
5.5	06_2i	92.0	92.4	94.7	100	100	100	70.0	70.0	75.0	ARI	1	0
9	07_6i	92.9	92.5	94.7	100	100	100	70.0	70.0	75.0	FLU	0	2
7	09_2i	84.0	84.0	89.5	90.5	90.5	93.3	70.0	70.0	75.0	ARI<br>SER	1<br>0	0<br>1
10	09_10i	92.0	92.4	84.7	100	100	100	70.0	70.0	75.0	ARI	1	0
8	10_3i	92.9	92.5	94.7	100	100	100	60.0	67.1	75.0	DUL	0	2
Mean ± SEM (*n* = 10)		93.1 ± 1.82	93.1 ± 1.83	94.2 ± 1.62	97.2 ± 2.02	97.1 ± 2.07	98.0 ± 1.42	81.0 ± 5.26	81.7 ± 4.99	85.0 ± 4.08	NA	NA	NA

SVMs, support vector machines; model (e.g., 2i, 4 variables); you, Youden index; mcc, Matthews correlation coefficient; acc, accuracy; all, combined score for training and test sets; tra, training set; tes, test set; mis, misclassified drug; obs, observed class; pred, predicted class; classes: 0, antidepressant; 1, antipsychotic; 2, anxiolytic. NA, not applicable. Statistical scores are expressed as percentages and defined in the Methods. Each model was started with a different random seed number and a training:test ratio of 15:4 compounds. Test set partition: stratified by CLASS using random selection. Color codes: red, antidepressant; blue, antipsychotic; magenta, anxiolytic. The three-letter code names for the drugs are given in Table 1. The top-ranked model (shown in bold) used the following behavioral descriptors and relative sensitivities: NSS_08 (1.000), SHPH_12 (0.967), RSD_08 (0.937), random seed = 81819. Behavioral descriptor definitions are given in S7 Fig and Tables 2 and 3. The rank for each model was determined by applying the RANK.AVG function in Microsoft Excel 365 to SUM(training metrics + test metrics + (100×$D_{min)}$ /D)), where D_{min} = minimum number of descriptors, and D = number of descriptors.
