# Supplementary material for: Distinguishing classes of neuroactive drugs based on computational physicochemical properties and experimental phenotypic profiling in planarians
Source: PLoS One. 2025 Jan 30;20(1):e0315394. doi: 10.1371/journal.pone.0315394 (PMC11781733; doi:10.1371/journal.pone.0315394)
Supplement: S23 Table — (PDF) [file pone.0315394.s033.pdf]

S23 Table. Compiled normalized responses for assay controls.

Control type	Condition	CRO	STK	SHP	SHP1	SHP2	SHP3	SHP4	SHP5	SCR	PTX	ANX	RSD	RSB	SPD	SB1	SB2	LBT	NSS
Neg	100 µM ascorbic acid	0	0	0	0	0	0	0	0	0	4	0	0	1	-11	-13	-14	5	-17
Neg	100 µM ascorbic acid	0	8	8	0	0	0	0	8	13	0	-5	1	-8	9	-10	29	-1	-17
Neg	100 µM ascorbic acid	0	4	4	0	0	0	0	4	0	4	4	-7	-4	19	22	-18	-3	43
Neg	100 µM Sorbitol	0	0	0	0	0	0	0	0	0	21	4	1	1	4	-17	-9	1	0
Neg	100 µM Sorbitol	0	21	0	0	0	0	0	0	0	4	-21	34	17	-38	-60	-59	9	-19
Neg	100 µM Sorbitol	4	0	0	0	0	0	0	0	0	0	7	1	3	7	-7	-2	-1	19
Pos	3% DMSO	0	**35**	25	4	21	0	0	17	25	4	2	7	4	-56	-55	-56	16	-28
Pos	3% DMSO	8	**27**	9	0	0	0	0	9	32	9	3	-3	-5	7	0	30	8	-39
Pos	3% DMSO	0	**33**	0	4	0	0	0	0	21	29	11	2	2	-23	-32	-52	19	-6
Pos	1% ethanol	0	**30**	**83**	0	29	4	0	**54**	17	13	-9	27	13	**-62**	-75	-66	30	-30
Pos	1% ethanol	8	**52**	**74**	13	0	0	0	**65**	13	0	7	1	4	-27	-46	-33	14	-27
Pos	1% ethanol	13	**71**	**67**	4	0	0	0	**67**	33	0	16	-10	-11	16	63	1	-11	-24
Pos	1% SDS	0	25	0	0	0	4	0	0	4	8	-28	47	17	-43	-70	-49	3	-19
Pos	1% SDS	0	4	13	8	0	0	0	4	8	33	2	**91**	31	**-77**	**-91**	**-75**	6	-3
Pos	1% SDS	0	0	0	0	0	0	0	0	8	0	7	6	-4	11	-4	10	-3	-6

Bold values indicate significant responses outside of the range of the respective benchmark response for that endpoint. Endpoint abbreviations: CRO: crawl-out, STK: stickiness, SHP: body shape (numbers refer to distinct body shape classes. 1: contracted, 2: C-shape, 3: Corkscrew, 4: Pharynx extrusion, 5: Hyperkinesis), SCR: scrunching, PTX: phototaxis, ANX: anxiety, RSD: resting_dark, RSB: resting_blue, SPD: speed_dark, SB1: speed_blue1, SB2: speed_blue2, LBT: locomotor bursts_total; NSS: noxious stimuli_strength.
